# Cell-specific vulnerability to metabolic failure: the crucial role of parvalbumin expressing neurons in creatine transporter deficiency

**DOI:** 10.1186/s40478-023-01533-w

**Published:** 2023-03-07

**Authors:** Elsa Ghirardini, Giulia Sagona, Angel Marquez-Galera, Francesco Calugi, Carmen M. Navarron, Francesco Cacciante, Siwei Chen, Federica Di Vetta, Lorenzo Dadà, Raffaele Mazziotti, Leonardo Lupori, Elena Putignano, Pierre Baldi, Jose P. Lopez-Atalaya, Tommaso Pizzorusso, Laura Baroncelli

**Affiliations:** 1 Department of Developmental Neuroscience, IRCCS Stella Maris Foundation, Viale del Tirreno 331, 56128 Calambrone (PI), Italy; 2 Institute of Neuroscience, National Research Council (CNR), Via Giuseppe Moruzzi 1, 56124 Pisa, Italy; 3 Instituto de Neurociencias, Universidad Miguel Hernández - Consejo Superior de Investigaciones Científicas, Avenida Santiago Ramon Y Cajal, S/N, 03550 Sant Joan d’Alacant, Alicante, Spain; 4 Department of Neuroscience, Psychology, Drug Research and Child Health NEUROFARBA, University of Florence, Via Di San Salvi 12, 50135 Florence, Italy; 5 BIO@SNS Lab, Scuola Normale Superiore Di Pisa, Piazza Dei Cavalieri 7, 56126 Pisa, Italy; 6 Department of Computer Science and Institute for Genomics and Bioinformatics, University of California, Irvine, CA 92697-3435 USA

**Keywords:** Creatine transporter deficiency, Neurodevelopmental disorders, Parvalbumin neurons, Energy metabolism, Synapse

## Abstract

Mutations in the solute carrier family 6-member 8 (*Slc6a8*) gene, encoding the protein responsible for cellular creatine (Cr) uptake, cause Creatine Transporter Deficiency (CTD), an X-linked neurometabolic disorder presenting with intellectual disability, autistic-like features, and epilepsy. The pathological determinants of CTD are still poorly understood, hindering the development of therapies. In this study, we generated an extensive transcriptomic profile of CTD showing that Cr deficiency causes perturbations of gene expression in excitatory neurons, inhibitory cells, and oligodendrocytes which result in remodeling of circuit excitability and synaptic wiring. We also identified specific alterations of parvalbumin-expressing (PV^+^) interneurons, exhibiting a reduction in cellular and synaptic density, and a hypofunctional electrophysiological phenotype. Mice lacking *Slc6a8* only in PV^+^ interneurons recapitulated numerous CTD features, including cognitive deterioration, impaired cortical processing and hyperexcitability of brain circuits, demonstrating that Cr deficit in PV^+^ interneurons is sufficient to determine the neurological phenotype of CTD. Moreover, a pharmacological treatment targeted to restore the efficiency of PV^+^ synapses significantly improved cortical activity in *Slc6a8* knock-out animals. Altogether, these data demonstrate that *Slc6a8* is critical for the normal function of PV^+^ interneurons and that impairment of these cells is central in the disease pathogenesis, suggesting a novel therapeutic venue for CTD.

**Supplementary Information:**

The online version contains supplementary material available at 10.1186/s40478-023-01533-w.

## Introduction

Creatine Transporter Deficiency (CTD) is an X-linked metabolic disorder causing cerebral creatine (Cr) deficit, intellectual disability, psycho-motor impairment, autistic-like behavior, and epilepsy. CTD etiology has been related to multiple mutations in the solute carrier family 6-member 8 (*Slc6a8*) gene encoding the protein responsible for cellular Cr uptake [1, 2].

Animal models either lacking the *Slc6a8* gene or engineered to express an allele with a point mutation found in patients, largely reproduce the endophenotype of the human condition [3–8]. The availability of these transgenic lines of rodents allowed the scientific community to take initial steps towards dissecting the pathological determinants of CTD [8]. Loss-of-function of *Slc6a8* does not result in overt alterations of brain structure and neuronal density, but rather induces a subtle reorganization of cerebral circuits and cellular metabolic processes [3, 5, 7–15]. However, a clear picture of the key cellular players involved in the development and progression of CTD is still missing. This represents a major issue, because a better knowledge of the causative mechanisms is crucial to identify novel druggable targets of translational value for a disease that is still untreatable [8].

Different cell types in the brain have distinctive metabolic profiles [16], resulting in a highly diversified energy demand in neuronal and glial populations [17]. Neurons consume 75–80% of the energy produced [18, 19] and might be particularly susceptible to the decreased ATP availability observed in CTD [9]. Intriguingly, the analysis of Slc6a8 RNA and protein levels revealed that its expression presents a significant heterogeneity across brain circuits [20–22], with a prominent expression in fast-spiking, parvalbumin-expressing (PV^+^) interneurons and oligodendrocytes [22, 23]. Thus, we hypothesized that the perturbation of energy supply due to Cr deficiency might have a multiform impact on the different brain cell populations. To test this possibility, we used a multi-level approach, ranging from RNA sequencing to patch-clamp recordings, behavioral assessment, optical imaging and EEG to explore cell-specific consequences of *Slc6a8* deletion.

We found that a defective supply of Cr affects the transcriptome of excitatory and inhibitory neurons and oligodendrocytes, inducing a morphological and functional rearrangement of neural circuits. In this framework, we focused on the high-energy-requiring PV^+^ GABAergic interneurons, demonstrating that this cellular population crucially contributes to the pathogenesis of CTD and might represent a suitable target for therapeutic intervention.

## Materials and methods

### Animals

We employed male mice hemizygous for the deletion of exons 5–7 in the *Slc6a8* gene (KO; CrT^−/y^) and their wild-type (WT; CrT^+/y^) littermates [4]. To target patch-clamp recordings to PV^+^ interneurons, we injected an AAV pCAG-FLEX-EGFP-WPRE vector (Addgene viral prep #51502-AAV9) in the lateral ventricles of newborn CrT^+/y^ and CrT^−/y^ mice expressing Cre recombinase under the parvalbumin (PV) promoter (PV::CrT^+/y^ and PV::CrT^−/y^, respectively). Finally, we generated a conditional mouse line carrying the floxed *Slc6a8* and PV-Cre alleles (PV::CrTfl^+/y^ and PV::CrTfl^−/y^) to study the effects of cell-specific *Slc6a8* deletion. Mice carrying the deleted/floxed *Slc6a8* allele were on pure C57Bl/6 J background; mice with the PV-Cre allele were from the B6.129P2-Pvalbtm1(cre)Arbr/J strain (JAX stock #017320). Mutant and wild-type animals for each group were invariably selected from the same litters, with a minimum of three litters for each experiment. All experiments were authorized by the Italian Ministry of Health (#1052/2020-PR). Data collection and analysis were performed blind to experimental conditions. See Additional file 1 for a more detailed description of methods.

### Bulk RNA sequencing (bulk RNA-seq)

Total RNA was extracted from the cerebral cortices of 3 CrT^+/y^ and 4 CrT^−/y^ animals (postnatal day 100, PND100) using the Qiagen RNeasy Midi Kit (Qiagen Inc.). RNA libraries were constructed using the Universal Plus mRNA-Seq kit (Tecan Genomics) and sequenced on paired-end 150 bp mode on NovaSeq 6000 (Illumina) at the Istituto di Genomica Applicata (IGA). The resulting sequencing data for each library were post-processed to obtain FastQ files, then demultiplexed using Bcl2Fastq 2.20 version of the Illumina pipeline. FastQ files can be accessed at the GEO repository (GSE218797). Transcriptomic data were run through Limma [24] to determine differentially expressed genes (DEGs, adjusted p-value < 0.1). Gene Ontology (GO) analysis was performed using the ShinyGO tool [25]. To search for interaction networks of corresponding proteins, we used the STRING database [26]. Validation of top DEGs was performed with quantitative PCR (qPCR).

### Single-nucleus RNA sequencing (snRNA-seq)

Nuclei were acutely purified from the cerebral cortex of 2 CrT^+/y^ and 2 CrT^−/y^ animals (PND100) as described previously, with minor modifications [27]. The whole cortex of the right cerebral hemisphere was manually dissected for each mouse. Tissue dissection was performed with extreme caution to avoid cross contamination with underlying brain tissue. For every sample, 15000 nuclei were loaded into a Chromium Single Cell A Chip (10 × Genomics) and processed following the manufacturer’s instructions. Single-nuclei RNA-seq libraries were prepared using the Chromium Single Cell 3’ Library & Gel Bead kit v2 and i7 Multiplex kit (10 × Genomics). Pooled libraries were then loaded on a HiSeq2500 instrument (Illumina) and sequenced to obtain 75 bp paired-end reads. snRNA-seq data can be accessed at the GEO repository (GSE216766). Sequenced samples were processed using the Cell Ranger (v.3.1.0) pipeline (10 × Genomics). Downstream analyses were performed using the R package Seurat (v3.1.4). To explore the heterogeneity of the cortex, we identified 7 major cell populations. DEGs between the two groups were identified using the Wilcoxon Rank Sum test and Bonferroni correction (adjusted p-value < 0.1). GO analysis for each differentially expressed gene list was performed as described above. The overlap between snRNA-seq and bulk RNA-seq data was assessed using a Fisher Exact Test.

### Electrophysiology

Brain slices containing the prefrontal cortex were prepared from PND30-P40 animals as described [28]. A subset of experiments was performed on visually identified pyramidal cells of layer II/III from CrT^+/y^ (n = 8) and CrT^−/y^ (n = 5) mice. Recordings from PV^+^ interneurons of cortical layers II/III and V were obtained by targeting GFP positive cells in PV::CrT^+/y^ (n = 5) and PV::CrT^−/y^ (n = 8) animals injected with the AAV pCAG-FLEX-EGFP-WPRE vector. Patch pipettes (2.5–4.5 MΩ resistance) were made from borosilicate glass capillaries (World Precision Instruments) using a P-97 puller (Sutter Instruments) and filled with a potassium gluconate-based solution. Whole-cell recordings were acquired using a Multiclamp 700A controlled by Clampex 11.2 via a Digidata 1550B amplifier (Molecular Devices). Clampfit 10.7 software was used for analysis. Differences between the groups were assessed with either a two-tailed t-test or two-way ANOVA (Graphpad Prism 9.4.1).

### Immunohistochemistry

Coronal brain Sects. (45 μm) from CrT^+/y^ (n = 6) and 6 CrT^−/y^ (*n* = 6) mice were processed for immunohistochemistry (Parvalbumin, 1:1000, catalog #195004, Synaptic System). To quantify the density of PV^+^ cells and synaptic puncta in the cerebral cortex we used a Zeiss microscope (Carl Zeiss). Differences between the two groups were assessed with a two-tailed t-test (Graphpad Prism 9.4.1).

### Y maze

PV::CrTfl^+/y^(n = 9) and PV::CrTfl^−/y^ (*n* = 8) mice (PND180) were allowed to explore a Y-shaped maze for a single trial of 8 min. Trials were video-recorded (Noldus Ethovision XT) for offline analysis. A triad was defined as a set of three consecutive arm entries, with each entry being into a different arm of the maze (e.g., A-B-C). The alternation percentage was calculated by dividing the number of triads by the number of possible alternations [5]. A two-tailed t-test revealed the difference between the two groups.

### Open field and object recognition test

PV::CrTfl^+/y^ (n = 5) and PV::CrTfl^−/y^ (*n* = 5) mice (PDN180) were allowed to explore a squared arena for a single trial of 10 min (day 1, open field). An area corresponding to the center of the arena was defined to assess emotional behavior. Total movements of animals and the time spent in the center area were automatically computed by Noldus Ethovision. For the object recognition test (ORT), two identical objects were placed in diagonally opposite corners of the same arena, approximately 15 cm from the walls, and mice were allowed 10 min to explore them (day 2, familiarization phase). The testing phase was performed 24 h after the familiarization phase (day 3). One of the familiar objects was replaced with a new one and mice were allowed to explore the two objects for 5 min. A discrimination index was computed as DI = (T_new_—T_old_)/(T_new_ + T_old_), where T_new_ is the time spent exploring the new object, and T_old_ is the time spent exploring the old one [5]. A two-tailed t-test was used to reveal the differences between the two groups.

### Intrinsic optical signal (IOS) imaging

Surgery and imaging sessions were performed as described [29], starting at least two days after the Y maze. A metal ring was affixed over the binocular visual cortex cortex of PV::CrTfl^+/y^ (*n* = 13) and PV::CrTfl^−/y^ (*n* = 12) animals and used to secure the animal under the objective. Images were visualized using a custom-made setup based on a Leica macroscope (Leica Z6 APO coupled with a Leica PanApo 2.0X; Leica Microsystems) and red-light LED illumination (630 nm). Visual stimuli were sinusoidal wave gratings (0.03 c/deg, 20 cd/m2, 90%, 4 Hz), generated using Matlab Psychtoolbox. A two-tailed t-test assessed differences between groups.

### EEG recordings

A two-channel head mount was implanted on the skull of PV::CrTfl^+/y^ (*n* = 18) and 18 PV::CrTfl^−/y^ (n = 18) mice, at least two days after IOS imaging. EEG was recorded using a preamplifier connected to a data acquisition system and Sirenia Software 1.7.9 (Pinnacle Technology). We evaluated spontaneous (baseline) cortical activity for 24 h, before assessing the effects of kainic acid (KA; intraperitoneal injection, 10 mg/kg). To quantify seizure episodes, we used Sirenia Seizure Pro 1.8.4 [29]. A two-tailed t-test and χ^2^ test were used to assess differences between groups.

### Stereotaxic injections of zolpidem

Zolpidem (100 μM) was injected at 3 sites surrounding the binocular visual cortex in CrT^+/y^ (n = 8) and CrT^−/y^ (n = 10) mice. A two-tailed t-test and two-way repeated measures ANOVA followed by post-hoc Holm–Sidak test were used to assess the effect of zolpidem treatment on cortical activity.

## Results

### Creatine deficiency affects brain expression of genes related to metabolism and synaptic signaling

**Fig. 1:**
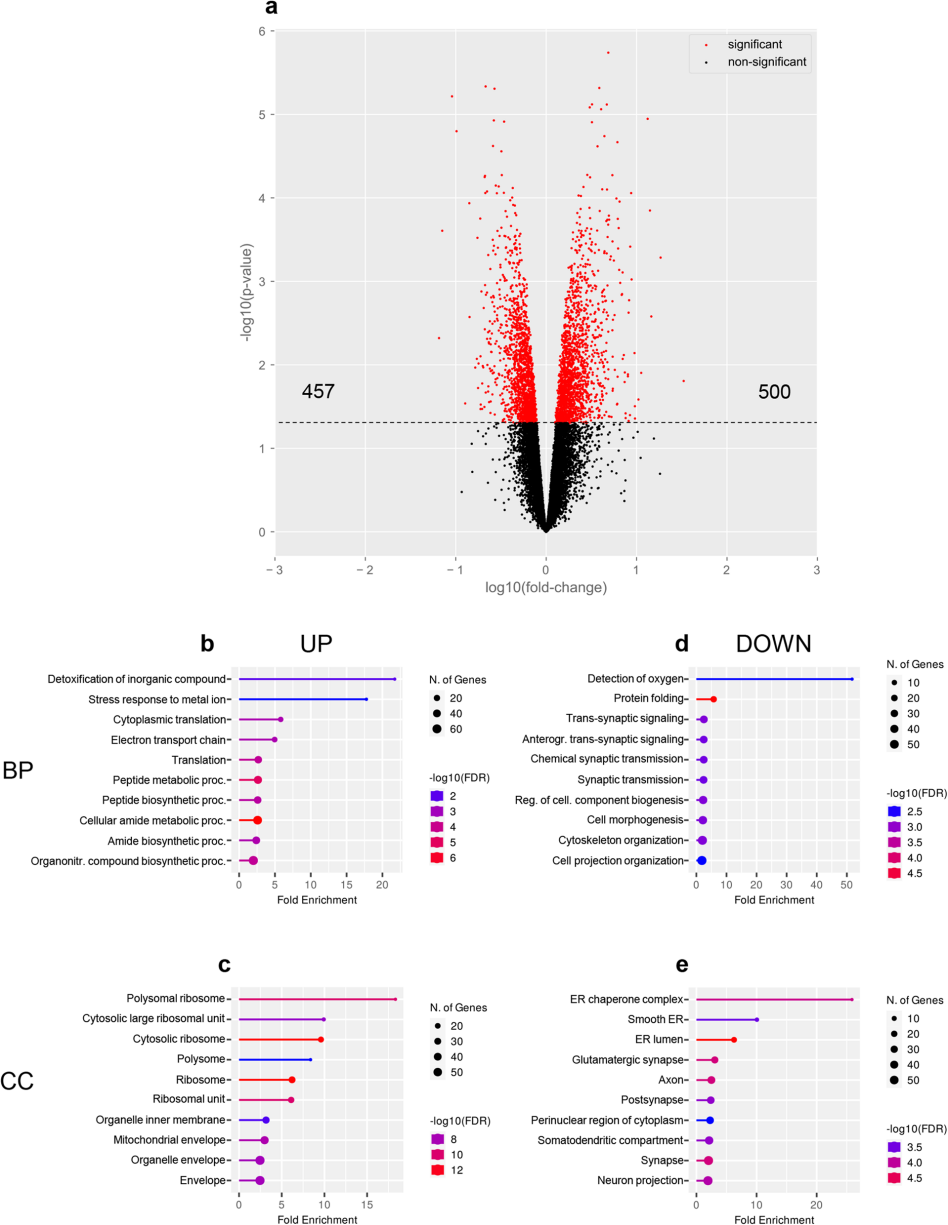
Differentially expressed genes in the cerebral cortex of CrT^−/y^ mice. **a** Volcano plot of the DEGs in the bulk RNA-seq data sets from CrT^+/y^ (*n* = 3) and CrT^−/y^ (*n* = 4) animals. Red points mark the significantly up- or downregulated genes in the CrT^−/y^ cerebral cortex (*p* < 0.1). **b**,**d** Fold enrichment of top-level overrepresented GO terms for biological process (BP) highlighting metabolic pathways in the set of upregulated genes (**b**) and synaptic pathways in the set of downregulated genes (**d**). **c,e** Fold enrichment of top-level overrepresented GO terms for cellular component (CC) highlighting ribosomes and mitochondria in the set of upregulated genes (**c**), and ER and synapses in the set of downregulated genes (**e**). Calculated by ShinyGO 0.76.3, FDR < 0.05

To investigate how gene expression is altered in conditions of Cr depletion, we performed bulk RNA-seq from the cerebral cortex of adult CrT^+/y^ and CrT^−/y^ animals. We found 957 genes which expression was regulated by Cr, with 500 genes upregulated and 457 genes downregulated (Fig. 1a; Additional file 2: Table S1). As expected, *Slc6a8* was significantly downregulated in the cortex of CrT^−/y^ mutant mice. These results were confirmed by qPCR (Additional file 3: Table S2). GO analysis for biological process revealed that the transcription of many genes involved in the cellular response to stress, protein translation and energy metabolism were upregulated (Fig. 1b; Additional file 4: Table S3), with ribosomes and mitochondria being the most enriched compartments in cellular component analysis (Fig. 1c; Additional file 5: Table S4). Accordingly, a search for corresponding protein–protein interaction (PPI) networks indicated that these genes were significantly connected in biological clusters related to the ribosomal function and the mitochondrial respiratory chain (Additional file 1: Fig. S1; Additional file 6: Table S5). In contrast, the list of downregulated genes was enriched for items implicated in the regulation of protein folding and synaptic signaling (Fig. 1d; Additional file 4: Table S3), and their transcription was primarily localized in the endoplasmic reticulum (ER) and postsynaptic compartment (Fig. 1e; Additional file 5: Table S4). PPI analysis identified the chaperone complex as the most represented cluster (Additional file 1: Fig. S2; Additional file 6: Table S5).

### Creatine deficiency affects the transcriptome of excitatory neurons, inhibitory cells and oligodendrocytes

**Fig. 2:**
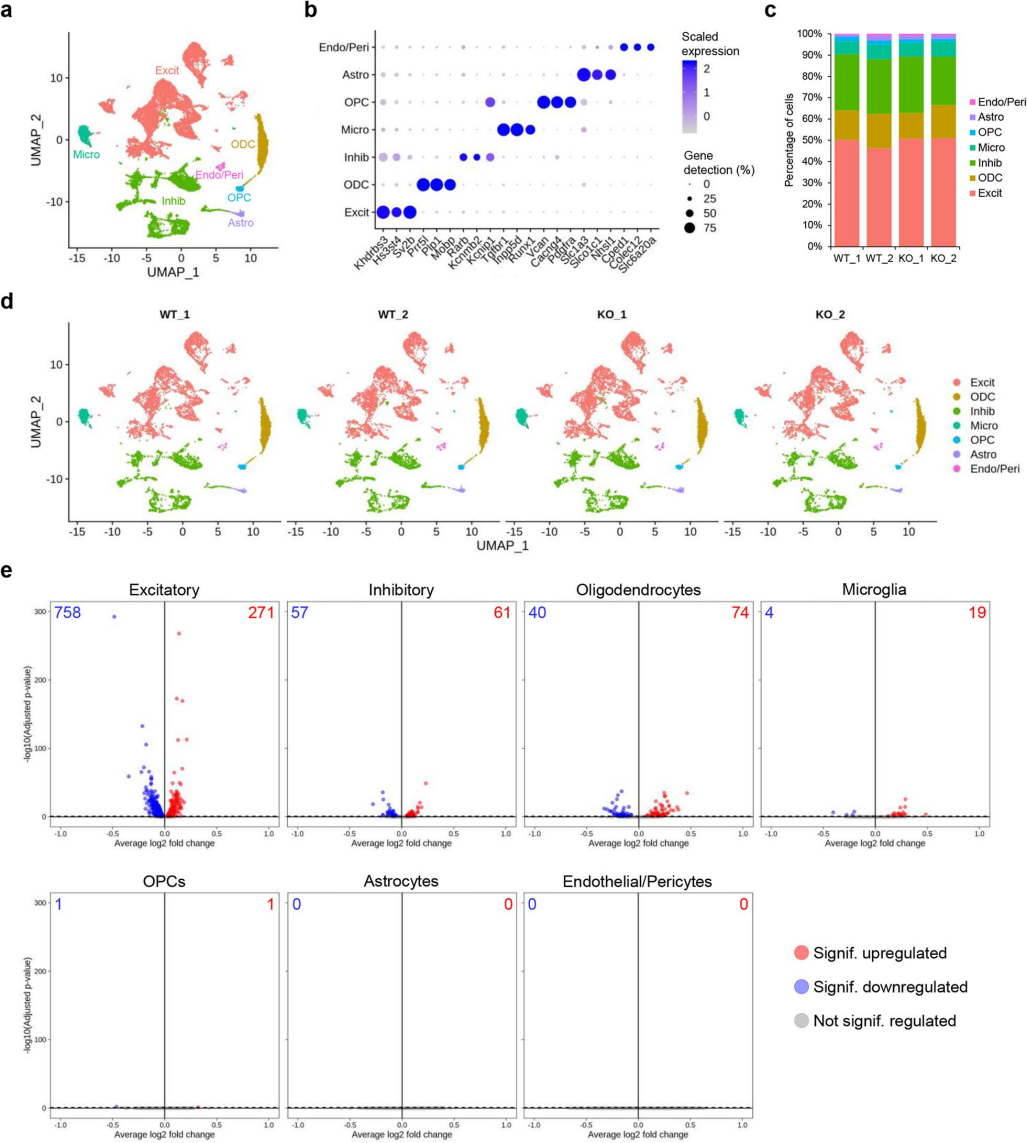
SnRNA-seq analysis of the cerebral cortex of CrT^+/y^ (WT) and CrT^−/y^ (KO) animals**. a** UMAP plot showing the populations identified in the snRNA-seq dataset of CrT^+/y^ (*n* = 2) and CrT^−/y^ (*n* = 2) animals. **b** Dot plot showing expression levels and gene detection of markers for the 7 major populations identified. **c** Bar graphs showing comparable distribution of each cell population between replicates and genotypes. **d** UMAP plot showing the populations identified in the snRNA-seq dataset of CrT^+/y^ and CrT^−/y^ animals by sample of origin. **e** Volcano plots showing the DEGs for each major cell population. The up- and downregulated genes are marked in red and blue, respectively, and their number is indicated at the top of each plot. Significant differences were detected in excitatory and inhibitory neurons, oligodendrocytes, and microglia, but not in astrocytes and vascular/endothelial cells (Wilcoxon Rank Sum test, adj p-value < 0.1)

We then used snRNA-seq to generate a comprehensive map of the most affected cell types in the brain of CrT^−/y^ mice. Supervised clustering analysis using canonical marker genes classified nuclei into seven major populations representing the main cell types: excitatory neurons, inhibitory neurons, astrocytes, oligodendrocytes (ODCs), oligodendrocyte precursors cells (OPCs), microglia, and vascular/endothelial cells (Fig. 2a,b; Additional file 1: Fig. S3d; Additional file 7: Table S6). Importantly, the *Slc6a8* gene was significantly downregulated in CrT^−/y^ mutant mice (Additional file 1: Fig. S3e,f). Integrative analysis did not detect any compositional differences between the datasets (Fig. 2a-d; Additional file 8: Table S7), indicating that Cr deficiency does not cause major changes in the proportion of specific cell populations.

Differential gene expression analysis revealed that 1146 genes were dynamically regulated across the genotypes in the cerebral cortex: while we failed to detect any differentially regulated genes (DEGs) in astrocytes and endothelial cells, we found significant changes in the transcriptome of excitatory (1029 genes: 271 upregulated and 758 downregulated) and inhibitory neurons (114 genes: 74 upregulated and 40 downregulated), oligodendrocytes (118 genes: 61 upregulated and 57 downregulated) and their precursors (OPCs, 2 genes: 1 upregulated and 1 downregulated), and microglial cells (23 genes: 19 upregulated and 4 downregulated; Fig. 2e, Additional file 9: Table S8). These data suggest that CTD affects multiple cell types, with neurons and oligodendrocytes being the most impacted populations. The validity of snRNA-seq was also strengthened by the following observations: (i) a significant overlap was present between the DEGs in excitatory neurons, the most represented cell type in single nucleus profiling, and those identified with bulk RNA-seq (Additional file 1: Fig. S4a); (ii) a significant correlation of the fold change was detected using snRNA-seq and bulk RNA-seq for DEGs common to the two lists (Additional file 1: Fig. S4b).

**Fig. 3:**
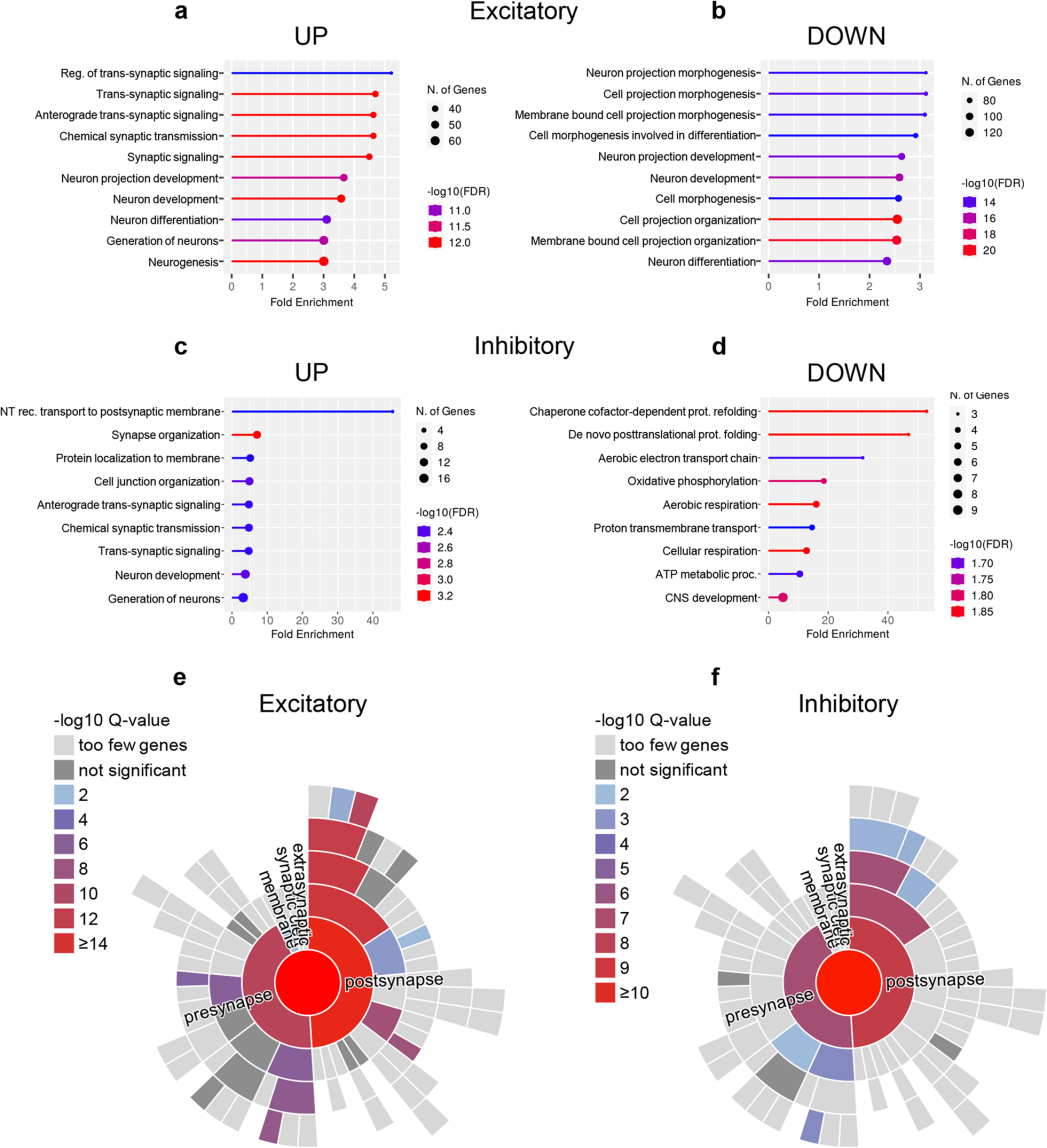
GO analysis for biological process from the snRNA-seq dataset in neuronal clusters. **a**-**d** Fold enrichment of top-level overrepresented GO terms in excitatory (**a**,**b**) and inhibitory (**c**,**d**) neurons from CrT^+/y^ (*n* = 2) and CrT^−/y^ (*n* = 2) animals showing enrichment in synaptic pathways. Calculated by ShinyGO 0.76.3, FDR < 0.05. **e,f** Hierarchical dendrograms of synaptic proteins corresponding to DEGs (SynGO database) identified in the same animals showing significant enrichment by color code: (**e**) excitatory neurons (**f**) inhibitory neurons

**Fig. 4:**
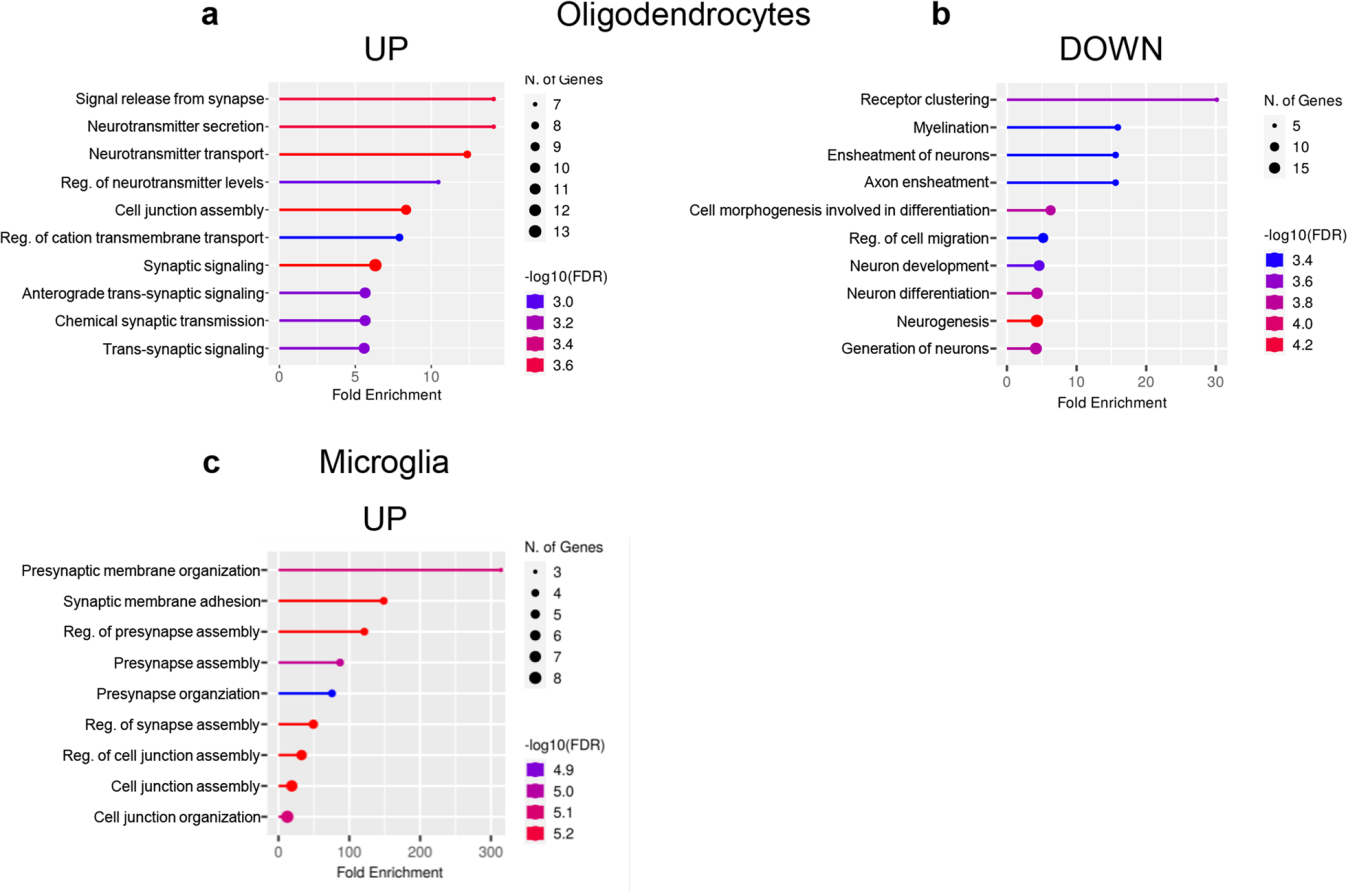
GO analysis for biological process from the snRNA-seq dataset in non-neuronal clusters**.** Fold enrichment of top-level overrepresented GO terms in oligodendrocytes **(a)** and microglia **(c)** from CrT^+/y^ (*n* = 2) and CrT^−/y^ (*n* = 2) animals showing enrichment in synaptic pathways among the upregulated genes. **b** Fold enrichment of top-level overrepresented GO terms in oligodendrocytes showing enrichment in myelin and axonal pathways among the downregulated genes. Calculated by ShinyGO 0.76.3, FDR < 0.05

GO analysis revealed that genes associated with synaptic assembly, neurotransmission and circuit development were enriched for dynamic expression across the genotypes in both excitatory (Fig. 3a, b; Additional file 1: Fig. S5a,b) and inhibitory neurons (Fig. 3c, d; Additional file 1: Fig. S5c,d; Additional file 10: Table S9; Additional file 11: Table S10), suggesting that neurological symptoms of CTD might primarily stem from subtle defects of subcellular compartments such as dendrites, axons or synapses. While the alteration of these functional modules was bidirectional in excitatory neurons (Fig. 3a, b), overrepresentation of synaptic pathways was specific to the upregulated genes of inhibitory neurons. In contrast, this population was characterized by reduced levels of metabolic and proteostatic genes (Fig. 3d; Additional file 10: Table S9; Additional file 11: Table S10). To get more specific insight into synaptic transcripts, we analyzed the lists of neuronal DEGs using SynGO, a curated database of proteins involved in synaptic functions and plasticity [30]. A significant overrepresentation of genes encoding for pre- and postsynaptic proteins was present in both excitatory (181/1029) and inhibitory neurons (34/114; Fig. 3e, f). Interestingly, the upregulation of synaptic-related items was also detected in oligodendrocytes and microglia (Fig. 4; Additional file 10: Table S9; Additional file 11: Table S10). Moreover, a decreased expression of genes involved in myelination processes was observed in CrT^−/y^ oligodendrocytes (Fig. 4b; Additional file 10: Table S9; Additional file 11: Table S10). These data suggest that a subtle reorganization of brain circuits underlies the pathogenesis of CTD.

### Opposite effects of Cr deficiency on the functional output of pyramidal and parvalbumin neurons

**Fig. 5:**
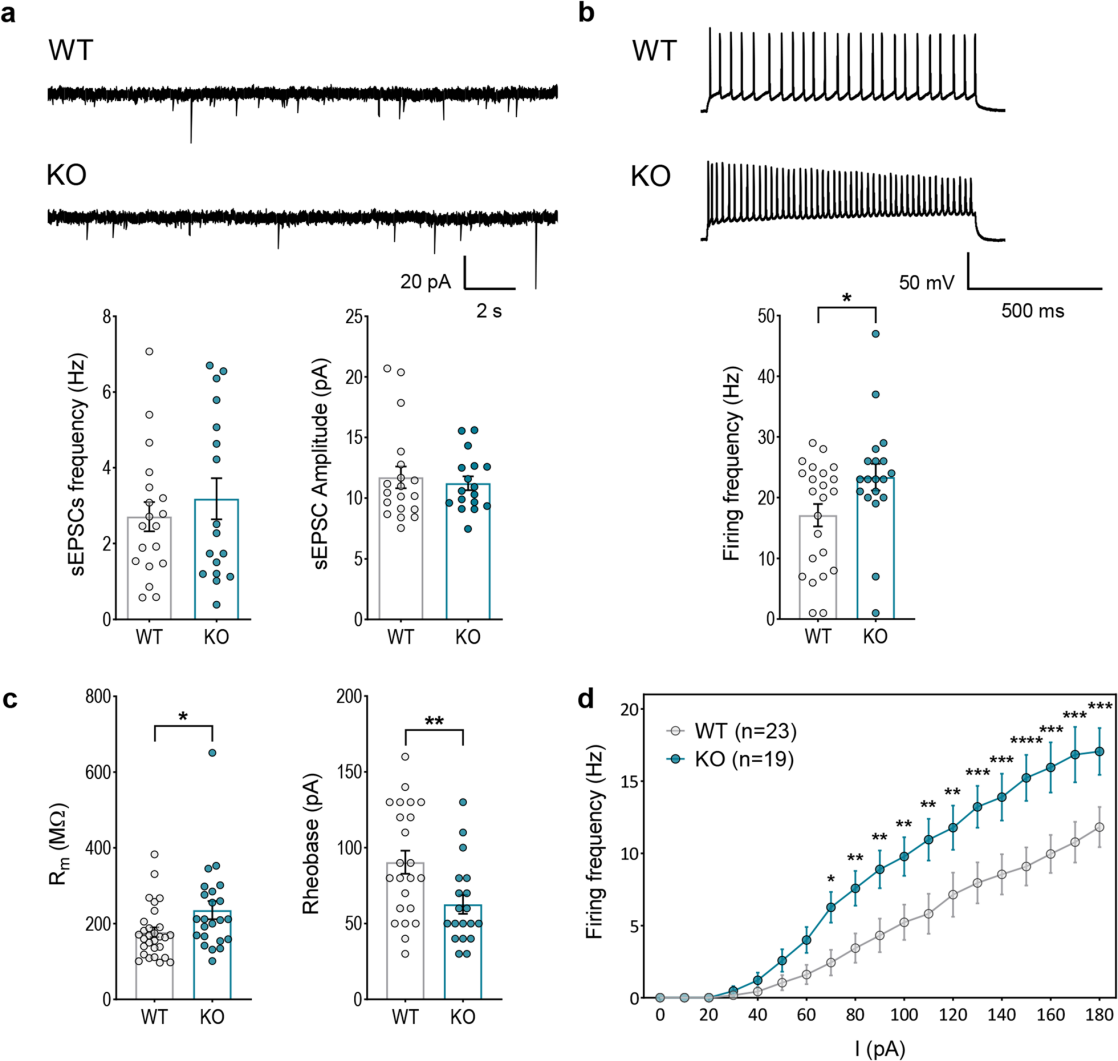
Electrophysiological characterization of pyramidal neurons in the PFC of CrT^+/y^ (WT) and CrT^−/y^ (KO) mice**.** Recordings were obtained from layer II/III pyramidal neurons from CrT^+/y^ (*n* = 8) and CrT^−/y^ (*n* = 5) animals at PND35-40, dots represent individual cells. **a** Representative traces (top) and quantification (bottom) of spontaneous excitatory postsynaptic currents (sEPSCs) in pyramidal cells of CrT^+/y^ and CrT^−/y^ animals. No differences were found between the genotypes in frequency (t-test, *p* = 0.477, *n* = 19 cells for CrT^+/y^ and 17 for CrT^−/y^) and amplitude (Mann–Whitney test, *p* = 0.851, *n* = 19 cells for CrT^+/y^ and 17 for CrT^−/y^). **b** Representative traces (top) and quantification (bottom) of firing frequency, showing an increased firing in CrT^−/y^ pyramidal neurons (t-test, *p* < 0.05, *n* = 23 cells for CrT^+/y^ and 19 for CrT^−/y^). **c** CrT^−/y^ pyramidal neurons display higher membrane resistance (left, Mann–Whitney test, p < 0.05, *n* = 29 cells for CrT^+/y^ and 22 for CrT^−/y^) and lower rheobase (right, t-test, *p* < 0.05, *n* = 23 cells for CrT^+/y^ and 19 for CrT^−/y^) compared to controls. **d** Frequency vs. current plot showing persistently increased firing frequency in CrT^−/y^ cells across a range of injected currents (two-way RM ANOVA followed by Fisher’s LSD test, *n* = 23 cells for CrT^+/y^ and 19 for CrT^−/y^). *p < 0.05, **p < 0.01, ***p < 0.001, ****p < 0.0001. Data are expressed as mean ± SEM

To investigate the functional consequences of *Slc6a8* deletion in specific neuronal populations, we first performed patch-clamp recordings in cortical pyramidal neurons. While we did not find any alterations in spontaneous synaptic activity (Fig. 5a; Additional file 1: Fig. S6a), we observed a significant increase in the firing frequency of pyramidal neurons in the cortex of CrT^−/y^ animals (Fig. 5b; Additional file 1: Fig. S6b). Consistently, we also found a significant rise of membrane resistance and a reduction of rheobase (Fig. 5c; Additional file 1: Fig. S6c). In addition, the firing pattern of mutant neurons in response to stimuli of increasing intensity was consistently more sustained compared to that of wild-type cells (Fig. 5d; Additional file 1: Fig. S6d).

**Fig. 6:**
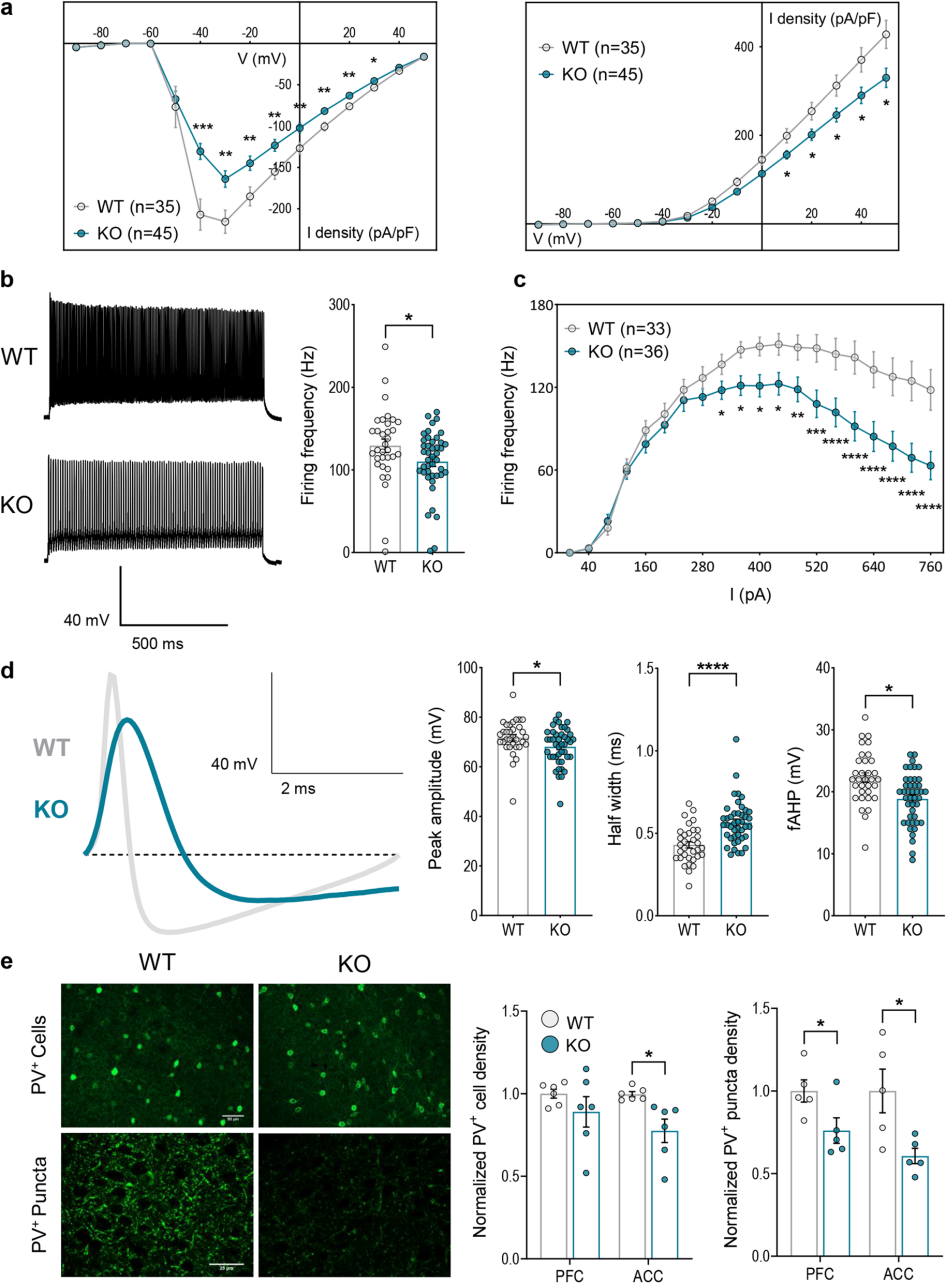
Characterization of PV^+^ interneurons in PFC and ACC of CrT^+/y^ (WT) and CrT^−/y^ (KO) mice**. a-d** Electrophysiological characterization of PV^+^ interneurons obtained from CrT^+/y^ and CrT^−/y^ animals, carrying the Cre-recombinase in PV^+^ interneurons (PV::CrT^+/y^, n = 5, and PV::CrT^−/y^, n = 8), injected with the AAV9 pCAG-FLEX-EGFP-WPRE vector; dots represent individual cells. **a** Current–voltage relationships showing a decrease in peak Na^+^ (left) and steady-state K^+^ (right) current densities in PV::CrT^−/y^ (KO) neurons with respect to controls (two-way RM ANOVA followed by Fisher’s LSD test, n = 35 cells for PV::CrT^+/y^ and 45 for PV::CrT^−/y^). **b** Representative traces (left) and quantification (right), showing a decrease in firing frequency in PV::CrT^−/y^ neurons (Mann–Whitney test, p < 0.05, n = 33 cells for PV::CrT^+/y^ and 43 for PV::CrT^−/y^). **c** Frequency vs. current plot showing that PV::CrT^−/y^ neurons persistently fire at lower frequency across a broad range of injected currents compared to controls (two-way RM ANOVA followed by Fisher’s LSD test, n = 33 cells for PV::CrT^+/y^ and 36 for PV::CrT^−/y^). **d** Representative trace showing the typical profile of action potentials in the two groups (left) and quantification of action potential amplitude (left graph), half-width (central graph) and fAHP (right graph), indicating an alteration of all three parameters in CrT^−/y^ neurons (Mann–Whitney test, p < 0.05 for peak amplitude and fAHP, *p* < 0.0001 for half-width, n = 33 cells for PV::CrT^+/y^ and 43 for PV::CrT^−/y^). **e** Representative images (left) and quantification of PV^+^ cells (center) and puncta density (right) in the PFC and ACC of CrT^+/y^ (*n* = 6) and CrT^−/y^ animals (n = 6), showing a global reduction in PV^+^ puncta and a decrease in PV^+^ cells restricted to the ACC (t-test, p < 0.05 for all comparisons). Dots represent individual animals. *p < 0.05, **p < 0.01, ***p < 0.001, ****p < 0.0001. Data are expressed as mean ± SEM

Since previous data indicated a specific alteration of the inhibitory phenotype in CTD, consisting in a decrease of the number of GABAergic, but not glutamatergic, synapses in the cerebral cortex of CrT^−/y^ mice [5], we also analyzed the contribution of inhibitory cells to CTD pathogenesis. Inhibition in the brain is mediated by a rich variety of GABAergic interneurons [31]. We focused on PV^+^ interneurons because they play a central role in controlling the spike timing of principal cells [32], their typical fast-spiking activity is highly energy-demanding [33], and they show prominent expression of *Slc6a8* [23]. Patch-clamp recordings of cortical PV^+^ interneurons showed a reduction of sodium and potassium voltage-gated currents (Fig. 6a; Additional file 1: Fig. S7a), a decline in firing frequency (Fig. 6b; Additional file 1: Fig. S7b), a reduced resistance to fatigue (Fig. 6c; Additional file 1: Fig. S7c), as well as an alteration of action potential amplitude, half-width and fast afterhyperpolarization (fAHP; Fig. 6d; Additional file 1: Fig. S7d) in CrT^−/y^ neurons. To investigate whether PV^+^ interneurons were also affected at the synaptic level, we used immunostaining for PV^+^ synaptic puncta. We found that PV^+^ synaptic density was decreased in the prefrontal cortex (PFC) and in the anterior cingulate cortex (ACC) of CrT^−/y^ mice. A reduced number of PV^+^ cells was also observed in the ACC (Fig. 6e). Overall, these data reveal a substantial impact of Cr deficiency on PV^+^ interneurons, emerging at both the morphological and functional level.

### Neurological endophenotype of conditional mice carrying a specific deletion of ***Slc6a8*** in PV^+^ interneurons

**Fig. 7:**
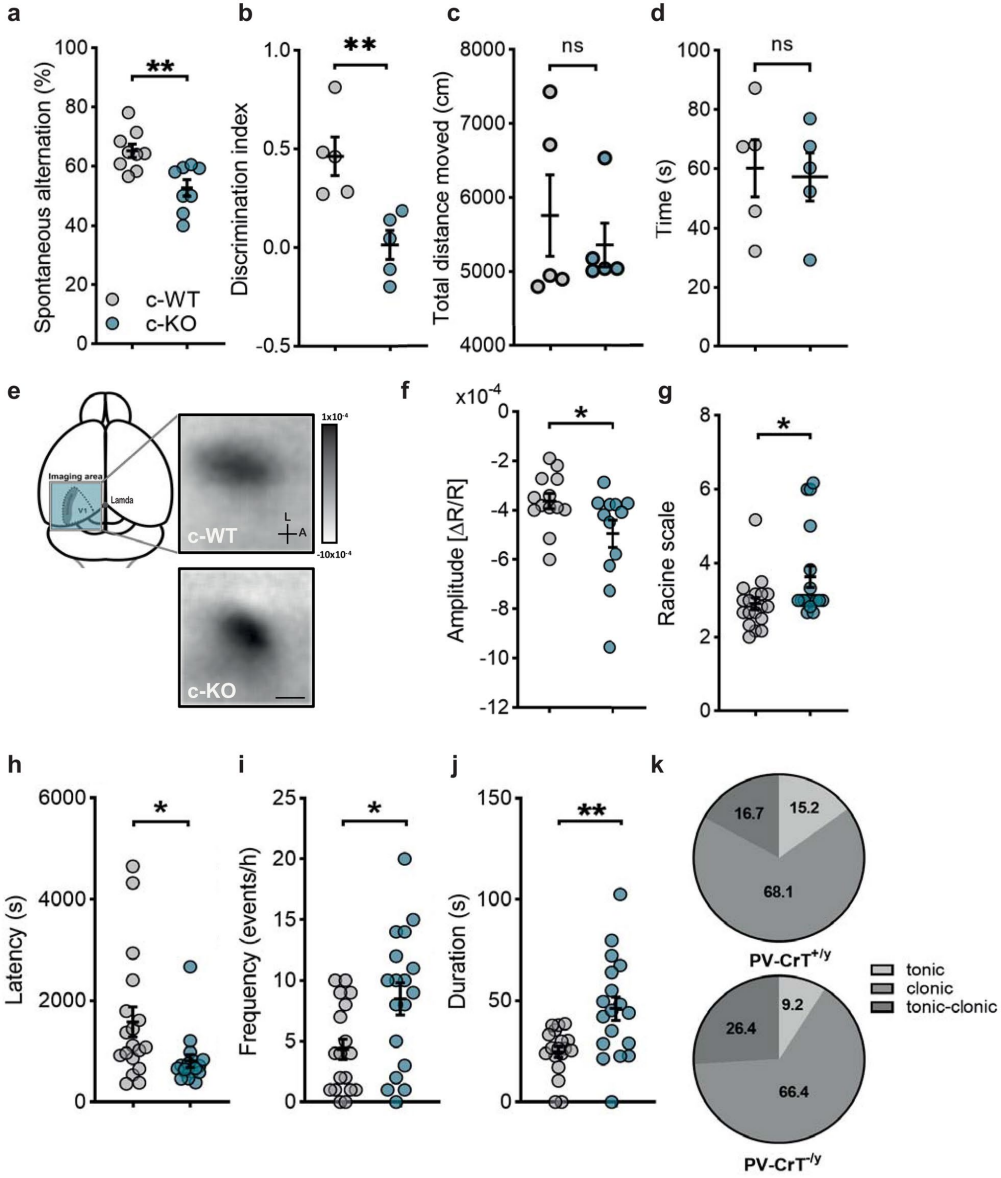
Behavioral, physiological and EEG characterization of PV::CrTfl animals. **a** Percentage of spontaneous alternation in the Y-maze test, showing a poorer performance in PV::CrTfl^−/y^ (c-KO, *n* = 8) mice compared to PV::CrTfl^+/y^ littermates (c-WT, *n* = 9; t-test, *p* < 0.01). **b** A significantly lower discrimination index was found in PV::CrTfl^−/y^ mice with respect to PV::CrTfl^+/y^ littermates (*n* = 5 for both groups; t-test p < 0.01). **c** Total distance moved in the open field arena did not differ between PV::CrTfl^−/y^ and PV::CrTfl^+/y^ mice (*n* = 5 for both groups; Mann–Whitney test, p = 0.69). **d** PV::CrTfl^−/y^ and PV::CrTfl^+/y^ animals spent a comparable amount of time in the center of the open field arena (t-test, *p* = 0.824). **e** Representative images of IOS imaging in the visual cortex of PV::CrTfl^+/y^ (top, *n* = 13) and PV::CrTfl^−/y^ mice (bottom, *n* = 12). Dark areas represent active portions of brain tissue. The look-up-table is also shown. Scale bar: 1.8 mm. L, lateral; A, anterior. **f** Quantification of IOS imaging showed an increased amplitude of the hemodynamic response in mutant animals (t-test: p < 0.05). **g** Effect of kainic acid (KA) treatment at the behavioral level. Circles represent the maximum seizure rating score of individual mice over a period of 1 h after KA administration. PV::CrTfl^−/y^ mice displayed a higher Racine score compared to wild-type littermates (*n* = 18 for both groups; t-test, p < 0.05). **h–k** Severity of the epileptic phenotype in response to KA at the electrophysiological level. PV::CrTfl^−/y^ mice have a lower latency to the first seizure (h, t-test, *p* < 0.05), and increased frequency (i, t-test, *p* < 0.05) and duration (j, t-test, *p* < 0.01) of seizure events with respect to PV::CrTfl^+/y^ animals. For PV::CrTfl^+/y^ animals not presenting seizures during the 1 h of monitoring, the observation was extended until the occurrence of the first electrographical burst to provide a latency value. Circles represent single data values. Relative percentage of tonic, clonic and tonic–clonic seizures in PV::CrTfl^+/y^ and PV::CrTfl^−/y^ (k) indicates that seizure severity is more pronounced i PV::CrTfl^−/y^ animals (χ^2^ test; p < 0.001). *p < 0.05, **p < 0.01, ns: not significant. Data are expressed as mean ± SEM

In light of the marked impairment of PV^+^ interneurons and synapses in the brain of CrT^−/y^ mice, we asked whether the deletion of *Slc6a8* only in these cells might be sufficient to recapitulate the phenotype of the whole-body knockout animals. To this purpose, we generated a conditional mouse model carrying a floxed *Slc6a8* allele and expressing Cre recombinase under the PV promoter. The body weight of PV::CrTfl^−/y^ mice was slightly lower than that measured for PV::CrTfl^+/y^ littermates (Additional file 1: Fig. S8). Since we previously showed that the performance in the Y maze can accurately discriminate between mutant and wild-type animals in our whole-body model of CTD [29], we first used this behavioral task to evaluate the cognitive phenotype in the conditional model and we found a clear impairment of spontaneous alternation in PV::CrTfl^−/y^ mice (Fig. 7a). We also detected a deterioration of object recognition memory: the discrimination index in the ORT at 24 h, indeed, was significantly lower in conditional mutant mice compared to PV::CrTfl^+/y^ littermates, indicating a reduced capacity to recall the familiar object (Fig. 7b). We then analyzed general activity and anxiety-related behavior of PV::CrTfl^−/y^ and PV::CrTfl^+/y^ animals in the open field arena used for cognitive assessment. We found no difference in the total distance moved (Fig. 7c) and time spent in the center of the arena (Fig. 7d) among the two groups, indicating that the difference in cognitive capacities are not due to changes in the ability to cope with stress in challenging conditions. To verify whether the main neurophysiological alterations described in CrT^−/y^ animals were also present in the conditional model, we measured visually-evoked hemodynamic responses using IOS, and the susceptibility to epilepsy in response to kainic acid challenge [29]. As previously reported for whole-body CrT^−/y^ mice [29], PV::CrTfl^−/y^ animals showed a significant alteration in the amplitude of cortical response to visual stimulation (Fig. 7e,f) and a higher susceptibility to kainic acid challenge according to the Racine scale (Fig. 7g), with lower latency of epileptiform activity (Fig. 7h), and increased frequency, duration and severity of epileptic episodes (Fig. 7i-k).

### Acute administration of zolpidem improves CTD phenotype

**Fig. 8:**
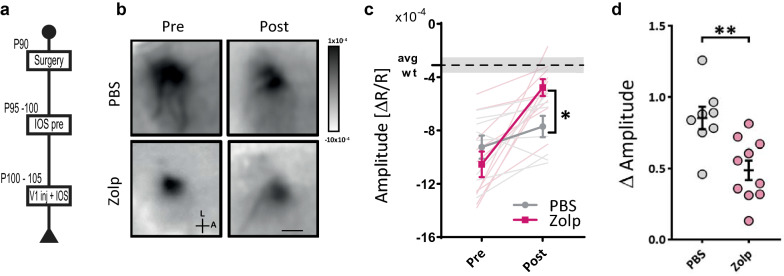
Visual IOS responses after zolpidem administration. **a** Experimental timeline. **b** Images and **c** quantification of IOS imaging in the visual cortex before and after zolpidem (*n* = 10, Zolp) or PBS injections, (*n* = 8) showing that Zolp treatment normalizes hemodynamic responses in CrT^−/y^ mice (two-way RM ANOVA, interaction time x treatment: p < 0.01, Sidak post-hoc multiple comparisons, p < 0.05 at post). Dashed line represents the average amplitude and grey area indicates the 95% confidence interval for WT mice, as previously measured [35]. The look-up-table is also shown. Scale bar: 1.8 mm. L, lateral; A, anterior. **c** Delta of pre-post injection amplitude (t-test, *p* < 0.01). *p < 0.05, **p < 0.01. Data are expressed as mean ± SEM. Circles represent single data values

Finally, we tested whether pharmacological manipulation of the activity of PV^+^ interneurons could rescue the CTD phenotype. Since it has been previously reported that GABA_A_ receptors incorporating the α1 subunit are enriched at the postsynaptic level in PV^+^ boutons [34], we used Zolpidem (Zolp), a specific agonist of α1-containing GABA_A_ receptors, to activate synaptic targets of PV^+^ interneurons [35, 36], and we measured IOS signals in the visual cortex of CrT^−/y^ animals before and after acute injection of Zolp or sterile PBS. We found that Zolp treatment significantly improved the amplitude of visually evoked IOS in CrT^−/y^ mice, whereas no significant change was detected upon vehicle injection (Fig. 8b,c,d). Notably, IOS amplitude in Zolp-treated CrT^−/y^ mice became comparable to that recorded in CrT^+/y^ animals (Fig. 8b).

## Discussion

Loss-of-function mutations in the *Slc6a8* gene cause a plethora of neuropsychiatric symptoms that are well reproduced in rodent models of CTD, indicating that cellular Cr uptake is critical for proper brain function and behavior [1, 8]. The Cr/phosphoCr system is a crucial hub for energy metabolism in every cell of the body, preserving ATP homeostasis and acting as an effective, mobilizable reservoir of high-energy phosphates generated in the mitochondrial compartment [37, 38]. The molecular pathophysiology of CTD involves a severe disruption of metabolism [9, 12, 39], resulting in the compensatory upregulation of proteins implicated in energy homeostasis and mitochondrial activity, which in turn exacerbates cellular oxidative stress [5, 6, 9, 10, 40].

A detailed analysis of the molecular mechanisms underlying the brain alterations produced by Cr depletion, however, is still missing. Using bulk RNA-seq, we had the opportunity to interrogate the genomic profile of the CTD brain and we found that key metabolic and cellular stress programs are upregulated in the cerebral cortex of CrT^−/y^ mice. These results corroborate the hypothesis that the inadequate supply of Cr in the brain overactivates energy-generating pathways in an attempt to compensate for the chronic power shortage, likely resulting in an overload of harmful by-products of cellular metabolism corrupting physiological processes [9, 10, 40]. Another intriguing and novel discovery is that *Slc6a8* deletion also downregulates the expression of genes involved in protein folding and synaptic signaling, suggesting that Cr deficiency might disrupt the function and maintenance of synaptic circuits [41].

Bulk RNA-seq measures the average gene expression across the variety of cellular populations present in the sample, providing an overview of global differences in the transcriptome of the brain between the two genotypes. Given the highly heterogeneous expression of *Slc6a8* in the brain [20–22], this approach is likely to obscure the diversified and unique impact of Cr deficiency on the transcriptional programs of various cell types. Thus, we harnessed the technology of snRNA-seq to get better insight into the complexity of the effects of Cr deficiency on different cell populations [42]. With this approach we ruled out major alterations in the cellular composition of the brain of CrT^−/y^ mice. This is consistent with our previous data showing that thickness and neuronal density are unaffected in the cerebral cortex of these animals [5], and with neuroimaging studies in children with CTD reporting only mild structural abnormalities [43–45]. However, we found that *Slc6a8* deletion dramatically affects gene expression in excitatory and inhibitory neurons, and oligodendrocytes. Although this observation could be limited by the number of libraries (two replicates in each group), it indicates that these cell populations might be the main players in CTD pathogenesis. Gene ontology analysis revealed that genes associated with synaptic assembly, neurotransmission and circuit development/preservation were mostly enriched for dynamic expression across the genotypes, suggesting that alterations in the dendritic, axonal and/or synaptic compartments may strongly contribute to the neurological phenotype of CTD. Intriguingly, a significant fraction of DEGs (almost the 20% for excitatory neurons, and 30% for inhibitory cells) mapped onto the list of presynaptic and postsynaptic proteins of the SynGO database [30], suggesting that CTD might be considered a synaptopathy.

Using neurophysiological recordings, we investigated more in-depth the functional deterioration of CTD cortical circuits. The atypically high firing frequency, elevated membrane resistance and reduced rheobase that we found in pyramidal neurons indicate a hyperexcitability of the excitatory circuits that might correlate to the epileptic phenotype of CrT^−/y^ mice [29]. In the absence of changes in spontaneous excitatory synaptic currents, these data suggest that this hyperexcitability might depend on altered intrinsic electrical properties of pyramidal neurons, including the membrane density and distribution of ionic conductance and receptors. However, the maintenance of a proper functional output is also influenced by inhibitory inputs. We previously reported a decrease in the number of GABAergic, but not glutamatergic, synapses in the cerebral cortex of CrT^−/y^ mice [5]. Here, we identified a more specific decrease in the number of PV^+^ synapses in the PFC and ACC, suggesting a dysfunction of this cell population in the CTD brain. Consistently, we found a significant hypofunction of PV^+^ interneurons in the PFC. Despite future studies are needed to map the morphological and functional alteration of PV^+^ synapses across multiple brain regions and throughout the different stages of CTD progression, this defective phenotype may indeed result in a global reduction of cortical inhibitory tone with potential disruption of neural circuitry efficiency [46]. One of the possible causes for the hypofunction of PV^+^ interneurons might be that a lack of readily available ATP due to Cr deficiency may interfere with the maintenance of physiological ionic concentrations in the cells. The sodium–potassium ATPase (Na^+^-K^+^ ATPase) is the main responsible for this process, and its activity is one of the major sources of energy consumption in neurons [47]. Not surprisingly, its expression is predominant in the PV^+^ subclass [48–50]. It is therefore reasonable to speculate that malfunction of the Na^+^-K^+^ ATPase due to energy shortage may hamper the generation of high-frequency action potentials typical of these neurons.

Remarkably, the dysfunction of PV^+^ interneurons is sufficient to reproduce the cognitive deterioration and the hyperexcitability of cerebral circuits that are hallmarks of CTD. Conditional mice with *Slc6a8* deficiency restricted to this cellular population recapitulate the cognitive deficits, altered hemodynamic responses and abnormal susceptibility to KA observed in whole-body CrT^−/y^ animals. The phenotypic similarity between the two murine models suggests that the depletion of Cr in PV^+^ interneurons alone is sufficient to perturb the neural networks globally, leading to a CTD-like disruption of brain physiological processes. This result is particularly remarkable considering that PV^+^ interneurons represent approximately the 8–10% of the total neuronal population [32] and adds to the body of literature that identify these cells as major contributors to the etiology of several neurodevelopmental and neuropsychiatric disorders [51–60].

We need to acknowledge, however, that the replication of neurological features in PV::CrTfl^−/y^ mice is not complete, because spontaneous epileptic seizures are not present in the conditional mouse model and the appearance of the behavioral phenotype is delayed to adult life. This suggests that a synergistic impairment of multiple cell types is necessary to generate the full portrait of CTD symptoms. Accordingly, snRNA-seq data highlighted that also non-neuronal cells exhibit significant gene expression changes. The high expression of *Slc6a8* in oligodendrocytes suggests that the pathophysiology of CTD might derive, at least partially, from a disruption of the tight metabolic coupling between neurons and oligodendrocytes. Despite their relatively low energy requirements, glial cells are strong Cr producers [61, 62] and have been suggested to supply Cr to neurons to maintain the ionic gradient across the axolemma, propagate action potentials, and transport molecules and organelles [63]. Moreover, the decreased expression of myelin-related genes in CrT^−/y^ oligodendrocytes suggests that myelin defects may contribute to the defective neuronal function in the CTD brain. Notably, recent studies showed that axon myelination is essential to the function of mature inhibitory circuits [64, 65]. Thus, an impairment of oligodendrocytes function might cooperate with the specific alteration of PV^+^ interneurons in the pathogenesis of CTD. Finally, it is also worth noting that terms identifying synaptic processes and assembly recurred in the GO lists of both oligodendrocytes and microglia. These cellular populations actively regulate synaptic refinement in the developing and adult mouse cortex [66–69], suggesting the possibility that an exaggerated synaptic pruning might undermine the solidity of brain circuits in CTD.

## Conclusions

In summary, our study demonstrates that CTD pathogenesis is likely to have a complex multicellular profile with a potential network of cell-autonomous and non-autonomous effects, but the dysfunction of PV^+^ interneurons is a crucial mediator of the CTD neurological phenotype. Pharmacological manipulation of PV^+^ synapses can improve cortical processing in CrT^−/y^ mice, indicating that therapeutic strategies selectively protecting PV^+^ interneurons should be explored to prevent and/or minimize their deterioration in CTD. Drugs targeting dysfunctional PV^+^ circuits are already available and have shown beneficial effects in other neuropsychiatric disorders such as schizophrenia, Fragile X syndrome and Rett syndrome [57, 70]. Our results can hopefully set the background for investigating the applicability of these compounds and, more in general, for drug repurposing for the treatment of CTD.

## Supplementary Information

**Additional file 1**:  Supplementary Materials and Methods and Supplementary figures S1, S2, S3, S4, S5, S6, S7, S8.

**Additional file 2:** Table S1. List of differentially expressed genes in bulk RNA-seq.

**Additional file 3:** Table S2. Quantitative PCR validation of top differentially-expressed genes.

**Additional file 4:** Table S3. Gene Ontology analysis for biological process for up- and downregulated genes in bulk RNA-seq.

**Additional file 5:** Table S4. Gene Ontology analysis for cellular component for up- and downregulated genes in bulk RNA-seq.

**Additional file 6:** Table S5. Clusters of protein-protein interactions (PPI) for the corresponding proteins of up- and downregulated genes in bulk RNA-seq.

**Additional file 7:** Table S6. List of population marker genes used for supervised clustering analysis of snRNA-seq data.

**Additional file 8:** Table S7. Quantification of cells belonging to each of the major population clusters in snRNA-seq data.

**Additional file 9:** Table S8.  List of differentially expressed genes in major sn-RNAseq clusters.

**Additional file 10:** Table S9. Gene Ontology analysis for biological process for up- and downregulated genes in major sn-RNAseq clusters.

**Additional file 11:** Table S10. Gene Ontology analysis for cellular component for up- and downregulated genes in major sn-RNAseq clusters.

**Additional file 12:** Table S11. Number of reads for each biological replicate in bulk RNA-seq.

**Additional file 13:** Table S12. Primer sequences for qPCR.

## Data Availability

The data supporting the conclusions of this article are present in the paper and the Supplementary Material. Raw data from RNA-seq experiments are available at the at the GEO repository GSE218797 for bulk sequencing and GSE216766 for snRNA sequencing. Requests for data and materials should be submitted to the last author.

## Abbreviations

AAV vector: Adeno-associated viral vector
ACC: Anterior cingulate cortex
BP: Biological process
CC: Cellular component
Cr: Creatine
CTD: Creatine transporter deficiency
DEGs: Differentially expressed genes
EEG: Electroencephalography
ER: Endoplasmic reticulum
fAHP: Fast afterhyperpolarization
GO: Gene ontology
IOS: Intrinsic optical signal
KA: Kaininc acid
KO: Knock-out
ODCs: Oligodendrocytes
OPCs: Oligodendrocyte-precursor cells
ORT: Object recognition test
PDN: Postnatal day
PFC: Prefrontal cortex
PPI: Protein–protein interaction
PV^+^: Parvalbumin-expressing
qPCR: Quantitative PCR
RNA-seq: RNA sequencing
sEPSCs: Spontaneous excitatory postsynaptic currents
snRNA-seq: Single nucleus RNA sequencing
WT: Wild-type
Zolp: Zolpidem
