# Supplementary material for: Cell-specific vulnerability to metabolic failure: the crucial role of parvalbumin expressing neurons in creatine transporter deficiency
Source: Acta Neuropathol Commun. 2023 Mar 7;11:34. doi: 10.1186/s40478-023-01533-w (PMC9990224; doi:10.1186/s40478-023-01533-w)
Supplement: Supplementary file 1 — Additional file 1: Supplementary Materials and Methods and Supplementary figures S1, S2, S3, S4, S5, S6, S7, S8. [file 40478_2023_1533_MOESM1_ESM.pdf]

Cell-specific vulnerability to metabolic failure: the crucial role of parvalbumin expressing neurons in Creatine Transporter Deficiency

Elsa Ghirardini[1,2*#], Giulia Sagona[2*], Angel Marquez-Galera[3], Francesco Calugi[4,5], Carmen M Navarron[3], Francesco Cacciante[5], Siwei Chen[6], Federica Di Vetta[2], Lorenzo Dadà[2], Raffaele Mazziotti[2,4], Leonardo Lupori[1], Elena Putignano[2], Pierre Baldi[6], Jose P Lopez-Atalaya[3], Tommaso Pizzorusso[2,5], Laura Baroncelli[1,2]

[1] Department of Developmental Neuroscience, IRCCS Stella Maris Foundation, viale del Tirreno 331, 56128 Calambrone (PI), Italy.
[2] Institute of Neuroscience, National Research Council (CNR), via Giuseppe Moruzzi 1, 56124, Pisa, Italy.
[3] Instituto de Neurociencias, Universidad Miguel Hernández - Consejo Superior de Investigaciones Científicas, Avenida Santiago Ramon y Cajal, s/n, 03550 Sant Joan d'Alacant, Alicante, Spain.
[4] Department of Neuroscience, Psychology, Drug Research and Child Health NEUROFARBA, University of Florence, via di San Salvi 12, 50135, Florence, Italy.
[5] BIO@SNS lab, Scuola Normale Superiore di Pisa, piazza dei Cavalieri 7, 56126 Pisa, Italy.
[6] Department of Computer Science and Institute for Genomics and Bioinformatics, University of California, Irvine, CA-92697-3435, USA.
*These authors equally contributed to this work.
[#]To whom correspondence should be addressed: elsa.ghirardini@in.cnr.it

Corresponding author:
Elsa Ghirardini
Institute of Neuroscience, National Research Council (CNR)
via Giuseppe Moruzzi 1, Pisa I-56124, Italy.
Email: elsa.ghirardini@in.cnr.it
Tel: +390 503 153199 Fax: +390 503 153220

Supplementary Materials and Methods

Animals

For patch-clamp recordings in parvalbumin-expressing (PV^+) interneurons we used a mouse expressing Cre recombinase under the parvalbumin (PV) promoter (PV^{cre}, JAX stock #017320; [1]): $CrT^{+/-}$ females were crossed with PV^{cre} male mice to generate a mouse line carrying the *Slc6a8* deletion and PV-Cre alleles. $CrT^{+/y}$ and $CrT^{-/y}$ mice carrying the Cre-recombinase in PV^+ interneurons (PV::$CrT^{+/y}$ and PV::$CrT^{-/y}$) were used for experimental procedures. PV^{cre} mice were also used to target the *Slc6a8* deletion to PV^+ interneurons: $CrT^{+/fl}$ females were crossed with PV^{cre} male mice to generate a mouse line carrying the floxed *Slc6a8* and PV-Cre alleles. Mice with two genotypes were used for behavioral, imaging and EEG analyses: mice carrying the PV specific deletion of *Slc6a8* (PV::$CrTfl^{-/y}$) and mice expressing Cre-recombinase but without the floxed allele (PV::$CrTfl^{+/y}$). The two genotypes were invariably obtained from the same litters.
We did not include in our analysis $CrT^{fl/y}$ mice not expressing the Cre recombinase, because we have previously shown that they do not present a hypomorphic phenotype and have normal Cr levels [2,3]. Animals were housed in IVC cages (Emerald-500, Tecniplast) and placed in a temperature-controlled room (20-22°C) under a 12-h light/dark cycle. Food (4RF25 GLP Certificate, Mucedola) and water were available *ad libitum*. Genotyping was performed by standard PCR, as previously described [2]. Primers for Cre recombinase expression were: mutant F: AAATGCTTCTGTCCGTTTGC; mutant R: ATGTTTAGCTGGCCCAAATG; wild-type F: CAGAGCAGGCATGGTGACTA; wild-type R: AGTACCAAGCAGGCAGGAGA. The amplicon size for the Cre allele was 163 bp (mutant) and 500 bp (wild-type). All experiments were carried out in accordance with the European Directive of 22 September 2010 (EU/63/2010).

Sample collection

Adult (postnatal day 100, PND100) $CrT^{+/y}$ and $CrT^{-/y}$ animals were used for RNA sequencing (RNA-seq) experiments. To prevent circadian effects, mice were sacrificed within the same time frame (10:00–12:00h; light phase). Brains were removed, and the cerebral cortex was dissected and immediately processed.

Bulk RNA sequencing

We obtained more than 600 million reads in total. The number of reads for each replicate is reported in Additional file 12: Table S11. The reads from each replicate experiment were aligned to the reference genome assembly mm10 and the corresponding transcriptome using the Tuxedo protocol [4]. Reads uniquely aligned to known exons or splice junctions extracted with no more than two mismatches were included in the transcriptome. On average, the read mapping rate was 81.67% (SD = 0.011), and the concordant pair alignment rate was 71.6% (SD = 0.017). Gene expression levels were directly computed from the read alignment results for each replicate. Standard FPKM (fragments per kilobase of exon per million mapped reads) values were extracted for each gene covered by the sequencing data and each replicate used in this study.

qPCR

500 ng RNA were reverse-transcribed with a Quantitect Reverse Transcription Kit (Qiagen) using random primers, and quantitative PCR was performed in 96-well Multiplate plates (Bio-Rad Laboratories) using Step One Plus Real-time PCR System and PowerUp Sybr Green Master Mix (Applied Biosystems). Reaction conditions were 50°C for 2 min, 95°C for 2 min, then 95°C for 15 s alternating with 60°C for 1 min for 40 cycles. Reverse transcriptions were run in triplicate for each sample and used as technical replicates for qPCR. A template-free negative control was always included. For a list of primer sequences see Additional file 13: Table S12.

Single-nucleus RNA sequencing

Nuclei isolation- Brains were immediately removed into ice cold HBBS 1x. The cerebral cortex of the right hemisphere was manually dissected and transferred into 1 mL of ice-cold MACS buffer (0.5% BSA, 2 mM EDTA, PBS 1x). Each cortex was then homogenized 12-15 times with the pestle of a dounce homogenizer (20404, Lab Unlimited). Cell suspensions were passed through a 40 μm cell strainer, collected in a 2 mL tube and centrifuged for 15 min (500G, 4ºC). The resulting cell pellets were resuspended in 1 mL of lysis buffer (10 mM Tris-HCL, 10 mM NaCl, 3 mM MgCl2, 0.1% IGEPAL) and transferred into a 15 mL tube containing 9 mL of cell lysis buffer. The cell suspension was kept 5 min on ice, inverting the tube to mix the suspension every 1 minute. Samples were then spun down at 500G for 30 min in a pre-chilled centrifuge. The nuclei pellet was resuspended in PBS 1x 1% BSA, RNase inhibitor 0.2 U/μL (3335399001, Merck Life Science) and 15000 nuclei were purified by flow cytometry in a BD FACS Aria III (BD Biosciences). The whole process was carried out at 4ºC. A total of four libraries were prepared, with two samples per condition ($CrT^{+/y}$ and $CrT^{-/y}$). *Single-nucleus RNA sequencing*- Purified intact nuclei were processed through all steps of 10x Genomics kits to generate stable cDNA libraries. Libraries were then sequenced on HiSeq2500 (Illumina) platform to obtain more than 900 million reads in total (WT1>240M; WT2>238M; KO1>300M; KO2>209M). *snRNA-seq initial analysis*- Quality control of sequenced reads was performed using FastQC (Babraham Institute). They were then processed using the Cell Ranger (v.3.1.0) pipeline and aligned to a custom "pre-mRNA" GRCm38 (mm10) mouse reference genome (Ensembl annotation v99). The custom reference was built for genes with biotype as protein_coding, lincRNA or antisense, and defined the entire gene body to be an exon. Barcodes with total unique molecular identifier (UMI) count >10% of the 99th percentile of the expected recovered cells were selected for further analysis. After this filtering step, we retained a total of 35694 cells (WT1: 8282 cells; WT2: 9295 cells; KO1: 9270 cells; KO2: 8847 cells) for subsequent analyses. In all four libraries, mean read pairs per cell was above 23000 (WT1: 29018; WT2: 25703; KO1: 32417; KO2: 23627). Confident mapping to exonic regions was higher than 81% for each library; median unique counts per cell were as follows: WT1: 5925; WT2: 6006; KO1: 6700; KO2: 5548; and median detected genes per cell: WT1: 2191; WT2: 2169; KO1: 2312; KO2: 2037. *Cell quality control, filtering, and integration process*- Downstream analyses, such as quality control, integration, normalization, shared nearest neighbor graph-based clustering, differential expression analysis and visualization were performed using the R package Seurat (v3.1.4) within R Statistical Computing Platform (v3.6.3). UMI count data from each sample was normalized following a regularized negative binomial regression with Seurat function *SCTransform* with default parameters. To integrate the four libraries, the top 3000 gene features were selected for anchors identification based on their redundant detection across samples (*SelectIntegrationFeatures*). *PrepSCTIntegration* was run to ensure that all necessary Pearson residuals had been calculated. Next, we identified integration anchors by performing Canonical Correlation Analysis with *FindIntegrationAnchors* (default parameters and "SCT" as normalization method) and datasets were integrated using the pre-computed anchorset using *IntegrateData* function. After initial quality filtering, more than 300 genes were detected per nucleus. The dataset of samples contained 35694 nuclei, with a median of 6031 transcripts (UMIs) and 2174 genes per nucleus (see Fig. S3A for quality control metrics). *Dimensionality reduction and cluster detection*- Principal Component Analysis (PCA) dimensionality reduction was performed using the Seurat function *RunPCA* with default parameters. A Shared Nearest Neighbor (SNN) Graph for the integrated dataset was built using *FindNeighbors* over top 30 Principal Components (PCs), and cell clusters were identified by a shared nearest neighbor (SNN) modularity optimization-based clustering algorithm with *FindClusters* function. To explore the heterogeneity of the cortex, a first cell clustering was performed at a very low resolution (0.03), leading to the identification of 8 clusters. After further exploration of cortex cell heterogeneity by a second cell clustering at the default resolution (0.8) of

the *SCTransform* integration workflow, the data were manually curated into 7 major cell populations (Excit: Excitatory cells, ODC: Oligodendrocytes, Inhib: Inhibitory cells, Micro: Microglia, OPC: Oligodendrocyte precursor cells, Astro: Astrocytes, Endo/Peri: Endotelial cells and/or pericytes). Uniform manifold approximation and projection (UMAP) [5] dimensional reduction technique was performed over top 30 PCs using the *RunUMAP* function. *Normalization and differential expression gene testing*- For expression plots and differentially expressed gene testing, cell expression was Log-Normalized using *NormalizeData* function with default parameter settings. Genes differentially expressed between the two genotypes were identified using the Wilcoxon Rank Sum test with the *FindMarkers* function (argument logfc.threshold = 0 and default values on the other parameters). P-value adjustment was performed using Bonferroni correction based on the total number of detected genes in the dataset. *Major populations markers heatmap*- We took the logistic regression gene testing with samples as latent variables without thresholds in average log fold-change over Log-Normalized expression for the 7 major populations. A total of 4866 enriched and statistically significant genes (positive log fold-change and adj p-value < 0.1) were sorted by descending average log fold-change. A proportional downsampling up to 2000 cells per population and top 10 genes from previous lists were used to plot the heatmap, showing scaled Log-Normalized expression. To generate the dot plot of gene expression and detection for the 7 major populations, 3 genes were selected for each population from the top 10 marker genes, showing scaled Log-Normalized expression.

AAV neonatal injections

Neonatal (PND1) PV::CrT$^{+/y}$ and PV::CrT$^{-/y}$ mice were cryoanestethized, and 1μl of AAV9 pCAG-FLEX-EGFP-WPRE vector (Addgene viral prep # 51502-AAV9, viral titer 1.0x10^{13}; 32; AAV-GFP) was injected through a glass capillary in the lateral ventricles. Injections were performed using the following coordinates: 1 mm rostral to lambda, ± 0.3 mm lateral to the midline, 2 mm ventral to the pial surface.

Electrophysiology

Mice were anesthetized with isoflurane (4%) and decapitated. Brains were rapidly removed and put in oxygenated ice-cold cutting solution containing (in mM): 92 N-Methyl-D-glucamine (NMDG), 2.5 KCl, 10 $NaHCO_3$, 1.25 NaH_2PO_4, 20 HEPES, 10 $MgSO_4$, 0.5 $CaCl_2$, 3 pyruvate, 3 myo inositol, 2 thiourea, 5 ascorbic acid, 25 glucose, 60 sucrose (pH 7.3, 310 mOsm). Coronal slices of the prefrontal cortex (270 µm thick) were obtained using a vibratome (VT1200 Leica Microsystems) and transferred at 34°C for 30 min, during which time NaCl was gradually added to a final concentration of 92 mM. Slices were allowed to equilibrate for at least 1 h in artificial cerebrospinal fluid (aCSF) containing (in mM): 92 NaCl, 2.5 KCl, 10 $NaHCO_3$, 1.25 NaH_2PO_4, 20 HEPES, 2 $MgSO_4$, 0.5 $CaCl_2$ 3 pyruvate, 3 myo inositol, 2 thiourea, 5 ascorbic acid, 25 glucose and 40 sucrose (pH 7.3, 310 mOsm). For recordings, slices were moved to the recording chamber and perfused with aCSF at 32 °C containing (in mM): 126 NaCl, 2.5 KCl, 10 $NaHCO_3$, 1.25 NaH_2PO_4, 15 HEPES, 1 $MgSO_4$, 2 $CaCl_2$, 12.5 glucose and 0.4 ascorbic acid. All solutions were constantly oxygenated. The capillary potassium gluconate-based solution contained (in mM): 10 KCl, 2 $MgCl_2$, 10 HEPES, 130 potassium gluconate, 1 ethylene glycol tetraacetic acid (EGTA), 4 Mg-ATP and 0.3 Tris-GTP. For recordings, data were sampled at 25 kHz and low-pass filtered at 10 kHz. Series resistance (10 to 50 MΩ) was always compensated and monitored for consistence during recording. Cells with leak currents > 200 pA and with a membrane resistance < 100 MΩ were excluded. Spontaneous excitatory postsynaptic currents (sEPSCs) were recorded holding the cells at -70 mV. Traces were low-pass filtered at 1 kHz, sEPSCs were selected through a template-based analysis, and amplitude and frequency were automatically calculated. Current-voltage relationships were obtained using voltage steps of -90 to +50 mV for 500 ms at a holding potential

of -70 mV. Action potentials were evoked using a single 1 second depolarizing current injection (200 pA) or repeated current steps of 10/40 pA. Firing frequency and kinetics were determined using a threshold-based method. Rheobase was estimated as the current value at which the first action potential was fired during a slowly rising current ramp. Fast afterhyperpolarization (fAHP) was calculated for the first action potential evoked during a 1 second depolarising current step and was measured by subtracting the spike threshold from the AHP peak within 5 ms after spike initiation.

Immunohistochemistry

$CrT^{+/y}$ and $CrT^{-/y}$ mice were perfused with 4% paraformaldehyde in phosphate buffer (0.1M, pH 7.4; PB). Brains were post-fixed for 2h and impregnated with 30% sucrose in PB. Coronal brain sections (45 μm) were cut on a freezing microtome and processed for immunohistochemistry. After a blocking step, free-floating slices were incubated overnight at 4°C in a solution of primary antibody (Parvalbumin, 1:1000, catalog #195004, Synaptic System) and antigen-antibody interaction was revealed with suitable Alexa Fluor-conjugated secondary antibodies (1:400, catalog #706-545-148, Jackson ImmunoResearch). Sections were then counterstained with Hoechst dye (1:500, catalog #94403, Merck Life Science), mounted on microscope slides and coverslipped using Vectashield mounting medium (Vector Laboratories). PV^+ cells were counted using a Zeiss Axiophot microscope (Carl Zeiss) with 20x magnification and the StereoInvestigator software (MicroBrightField). Sampling boxes (250x250 μm) were located in both superficial and deep layers of prefrontal cortex (PFC) and anterior cingulate cortex (ACC). Cell density was averaged from at least 6 counting boxes per animal. To quantify the density of PV^+ puncta, we imaged superficial and deep layers of PFC and ACC using a Zeiss laser scanning Apotome microscope equipped with 63x oil immersion objective (Carl Zeiss). For each section, we imaged serial optical sections at 0.33 μm intervals for a total of at least 15 optical sections (5 μm). 6 sections were analyzed for each animal (3 in superficial layers and 3 in deep layers). Maximum intensity projections (MIPs) were generated from the group of 5 consecutive sections yielding the higher mean pixel intensity. These MIPs were imported in ImageJ and quantified using the Puncta analyzer plugin [6]. The number of positive puncta was measured within the entire acquired area.

Y maze

The Y maze has three symmetrical arms at a 120° angle (26cm length, 10cm width, 15cm height). An arm entry was scored when all four limbs of the animal were within the arm [2].

Open field and object recognition test

The apparatus consisted of a squared arena (60 x 60 x30 cm) constructed in poly(vinyl chloride) with black walls and a white floor. First, mice received one session of 10-min duration in the empty arena (open field test, day 1). Animal position was continuously recorded by the video tracking system (Noldus Ethovision XT). In the recording software, an area corresponding to the center of the arena (a central square 30 x 30 cm), and a peripheral region (corresponding to the remaining portion of the square) were defined. The object recognition test (ORT) consisted of two phases: familiarization and testing phase. During the familiarization phase (day 2), two identical objects were placed in diagonally opposite corners of the arena, approximately 15 cm from the walls, and mice were allowed 10 min to explore the objects. The objects were made of plastic, metal, or glass material and were too heavy to be displaced by mice. The testing phase was performed 24h after the familiarization phase (day 3). One of the two familiar objects was replaced with a new one, while the other object was replaced by an identical copy. The objects were placed in the same locations as the previous ones. Mice were allowed to explore objects for 5 min. To avoid possible preferences for one of two objects, the choice of the new and old object and the position of the

new one were randomized among animals. The amount of time spent exploring each object (nose sniffing and head orientation within <1.0 cm) was recorded and evaluated by the experimenter blind to the mouse genotype. Arena and objects were cleaned with 10% ethanol between trials to stop the build-up of olfactory cues. Mice exploring the two objects for less than 5 s during the sample phase were excluded from testing [2].

Intrinsic optical signal (IOS) imaging

A metal ring was attached to the skull in correspondence of the binocular visual cortex and a drop of transparent nail polish was employed to improve the optical access. Mice were anesthetized with isoflurane (1-3%) and secured under the objective using a magnet mounted on an arduino-based imaging chamber. Imaging was performed using a custom Leica macroscope. Frames were acquired at 30 fps with a 512×512 pixels resolution. Visual stimuli were displayed on a screen placed 13 cm away from the eyes of the animals. Fluctuations of reflectance (R) for each pixel were computed as the normalized difference from the average baseline (ΔR/R). A region of interest (ROI) was identified on the mean image of the contralateral eye response by selecting the pixels in the lowest 30% ΔR/R of the range between the maximal and minimal intensity and mean evoked response was quantitatively estimated as the average intensity within the ROI over at least 80 trials [7].

EEG recordings

EEG electrodes were placed over the frontal and the occipital areas. Video-recordings were performed in parallel during the entire length of EEG assessment. Signals were recorded at 400 Hz sampling frequency. After kaininc acid (KA) treatment, animals were observed for 1h. The baseline period of each animal was used as a cutoff threshold (mean line length + 8xSD). Events in the 2-10 Hz frequency range, with a line length higher than the defined threshold, and lasting at least 10 seconds were identified as seizures. At the behavioral level, seizures were scored according to the Racine scale. Seizures of stage 1 and 2 were classified as tonic events, seizures of stage 3 were assigned to clonic events and seizures of stage 4, 5 and 6 were categorized as tonic-clonic events [7].

Stereotaxic injections of zolpidem

$CrT^{+/y}$ and $CrT^{-/y}$ mice (PND100-105) were anesthetized with isoflurane (1-3%) and fixed on a stereotaxic frame. After placing a small burr hole, a glass microcapillary (80 µm tip) was inserted into the visual cortex at two different depths (200 and 600 μm) to inject zolpidem (100 μM, 0.25 μl per site) or sterile PBS. Coordinates of injections were identified by previous IOS imaging of baseline visual responses.

Supplementary References

1. Hippenmeyer S, Vrieseling E, Sigrist M, Portmann T, Laengle C, Ladle DR, Arber S (2005): A developmental switch in the response of DRG neurons to ETS transcription factor signaling. *PLoS Biol* 3: e159.
2. Baroncelli L, Molinaro A, Cacciante F, Alessandrì MG, Napoli D, Putignano E, *et al.* (2016): A mouse model for creatine transporter deficiency reveals early onset cognitive impairment and neuropathology associated with brain aging. *Hum Mol Genet* 25: 4186–4200.
3. Molinaro A, Alessandrì MG, Putignano E, Leuzzi V, Cioni G, Baroncelli L, Pizzorusso T (2019): A Nervous System-Specific Model of Creatine Transporter Deficiency Recapitulates the Cognitive Endophenotype of the Disease: a Longitudinal Study. *Sci Rep* 9: 62.
4. Trapnell C, Roberts A, Goff L, Pertea G, Kim D, Kelley DR, *et al.* (2012): Differential gene and

transcript expression analysis of RNA-seq experiments with TopHat and Cufflinks. *Nat Protoc* 7: 562–578.
5. Becht E, McInnes L, Healy J, Dutertre C-A, Kwok IWH, Ng LG, *et al.* (2018): Dimensionality reduction for visualizing single-cell data using UMAP. *Nat Biotechnol*. https://doi.org/10.1038/nbt.4314
6. Ippolito DM, Eroglu C (2010): Quantifying synapses: an immunocytochemistry-based assay to quantify synapse number. *J Vis Exp*. https://doi.org/10.3791/2270
7. Mazziotti R, Cacciante F, Sagona G, Lupori L, Gennaro M, Putignano E, *et al.* (2020): Novel translational phenotypes and biomarkers for creatine transporter deficiency. *Brain Commun* 2: fcaa089.

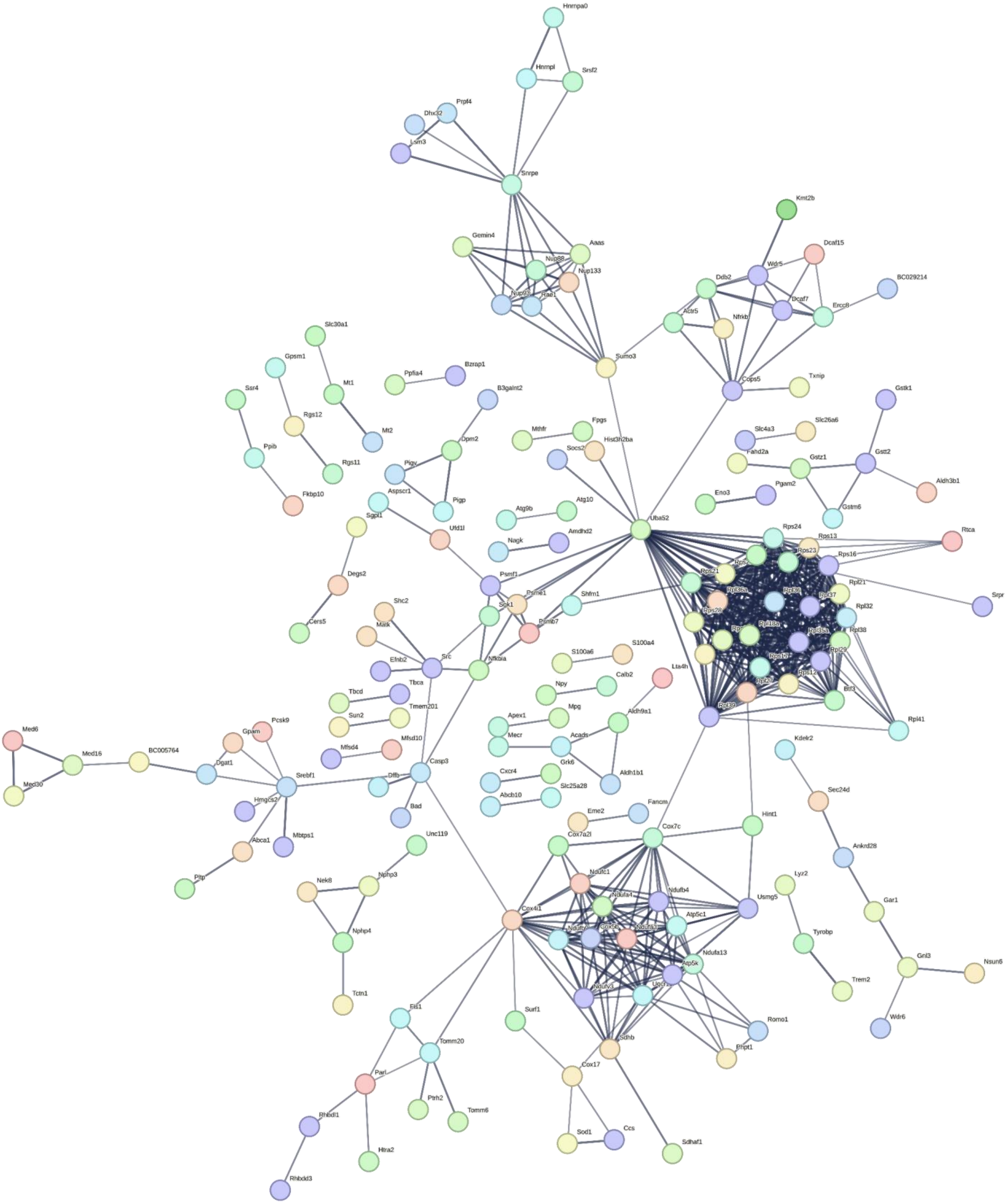

Figure S1. STRING protein-protein interactions (PPI) network of the proteins corresponding to upregulated genes identified with bulk RNA-seq. Genes were significantly connected in biological clusters related to the ribosomal function and the mitochondrial respiratory chain. Average local clustering coefficient 0.308; enrichment p-value 1.0e-16; average node degree 2.46.

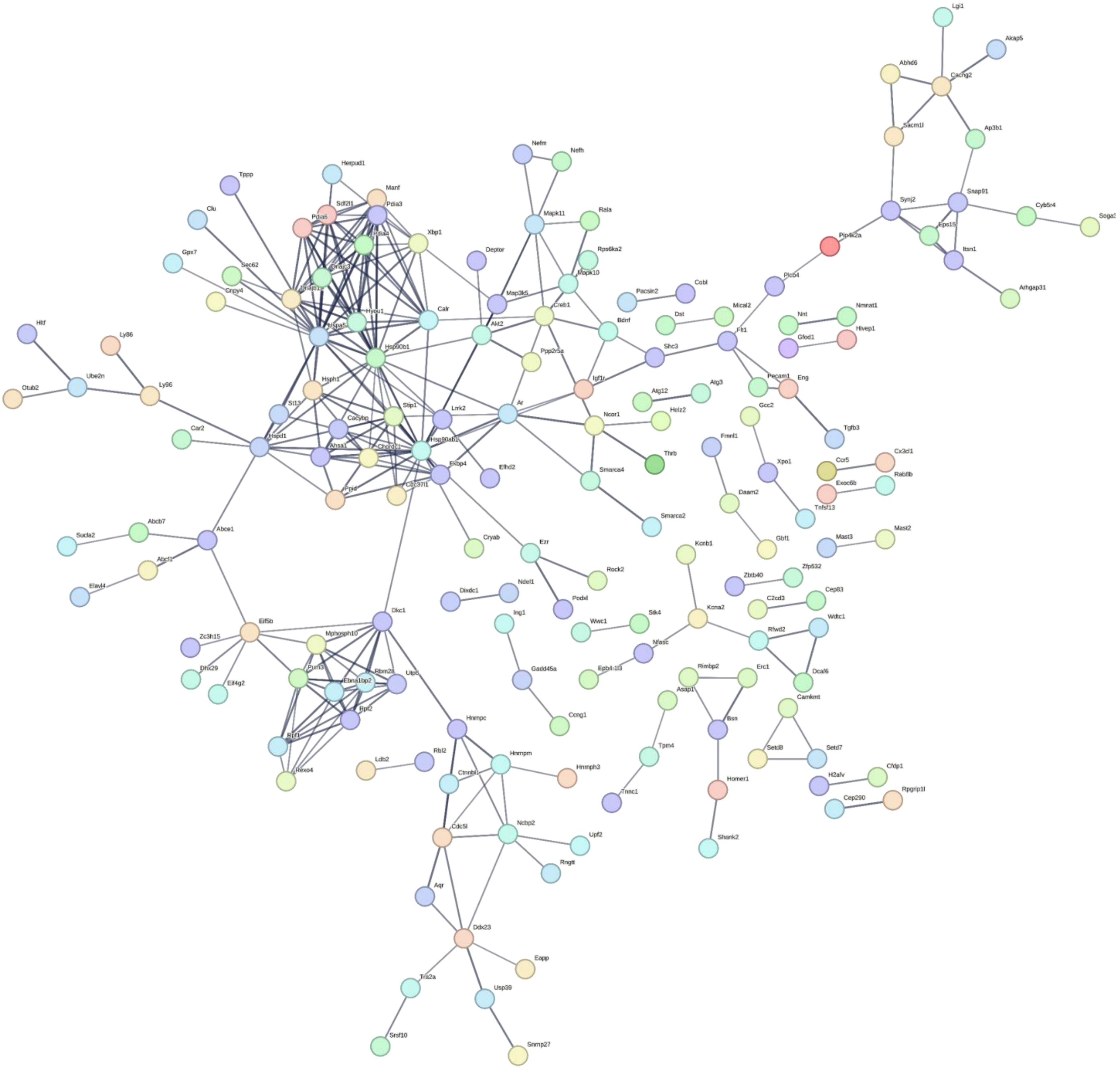

Figure S2. STRING PPI network of the proteins corresponding to downregulated genes identified with bulk RNA-seq. The chaperone complex is the most represented cluster. Average local clustering coefficient 0.278; enrichment p-value 1.0e-16; average node degree 1.35.

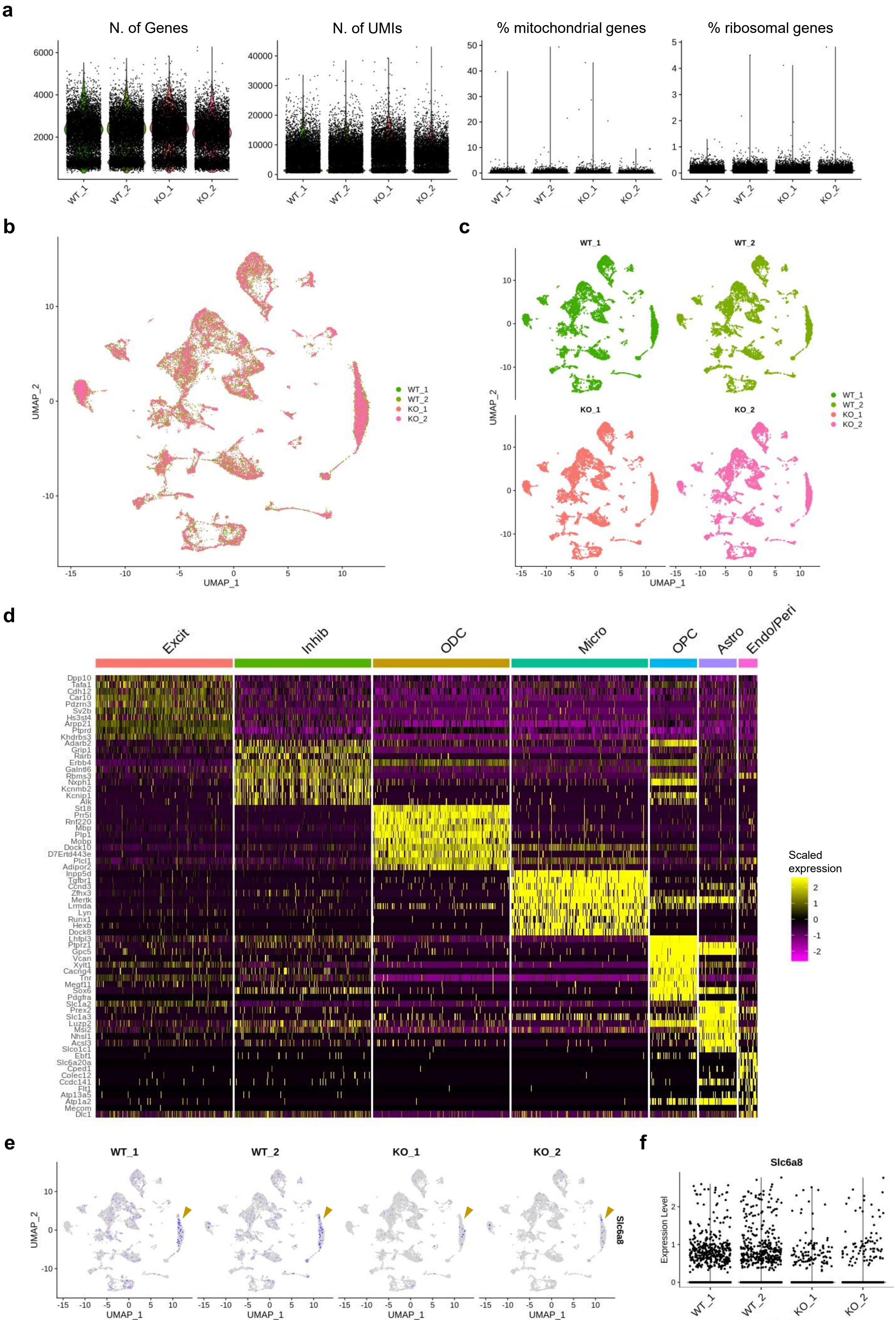
a
N. of Genes
N. of UMIs
% mitochondrial genes
% ribosomal genes
WT_1
WT_2
KO_1
KO_2
b
UMAP_1
UMAP_2
WT_1
WT_2
KO_1
KO_2
c
WT_1
WT_2
KO_1
KO_2
UMAP_1
UMAP_2
d
Excit
Inhib
ODC
Micro
OPC
Astro
Endo/Peri
Dpp10
Tafa1
Cdh12
Car10
Pdzrn3
Sv2b
Hs3st4
Arpp21
Ptprd
Khdrbs3
Adarb2
Grip1
Rarb
Erbb4
Galntl6
Rbms3
Nxph1
Kcnmb2
Kcnip1
Alk
St18
Prr5l
Rnf220
Mbp
Plp1
Mobp
Dock10
D7Ertd443e
Plcl1
Adipor2
Inpp5d
Tgfbr1
Ccnd3
Zfhx3
Mertk
Lrmda
Lyn
Runx1
Hexb
Dock8
Lhfpl3
Ptprz1
Gpc5
Vcan
Xylt1
Cacng4
Tnr
Megf11
Sox6
Pdgfra
Slc1a2
Prex2
Slc1a3
Luzp2
Msi2
Nhsl1
Acsl3
Slco1c1
Ebf1
Slc6a20a
Cped1
Colec12
Ccdc141
Flt1
Atp13a5
Atp1a2
Mecom
Dlc1
Scaled expression
e
WT_1
WT_2
KO_1
KO_2
UMAP_1
UMAP_2
Slc6a8
f
Slc6a8
Expression Level
WT_1
WT_2
KO_1
KO_2

Figure S3. snRNA-seq analysis of the cerebral cortex of CrT$^{+/y}$ and CrT$^{-/y}$ animals. a) Violin plots showing number of genes, number of UMIs, percentage of mitochondrial UMIs and percentage of ribosomal UMIs across the 4 samples. **b)** UMAP plot showing the overlay between the samples of snRNA-seq dataset of CrT$^{+/y}$ (n=2) and CrT$^{-/y}$ (n=2) animals. **c)** UMAP plot showing the cell distribution in the snRNA-seq dataset of CrT$^{+/y}$ (n=2) and CrT$^{-/y}$ (n=2) animals by sample of origin. **d)** Heatmap plot showing the top 10 markers for each major cell population (Logistic regression test with samples as latent variables, adj p-value < 0.1, top 10 markers by descending average log2 fold change). **e)** UMAP plot showing the *Slc6a8* normalized cell expression by sample of origin. Arrowheads point to ODC population. **f)** Violin plot showing *Slc6a8* normalized cell expression by sample of origin (log FC = -1.34; Wilcoxon rank sum test, adj p-value = 6.37×10^{-70}).

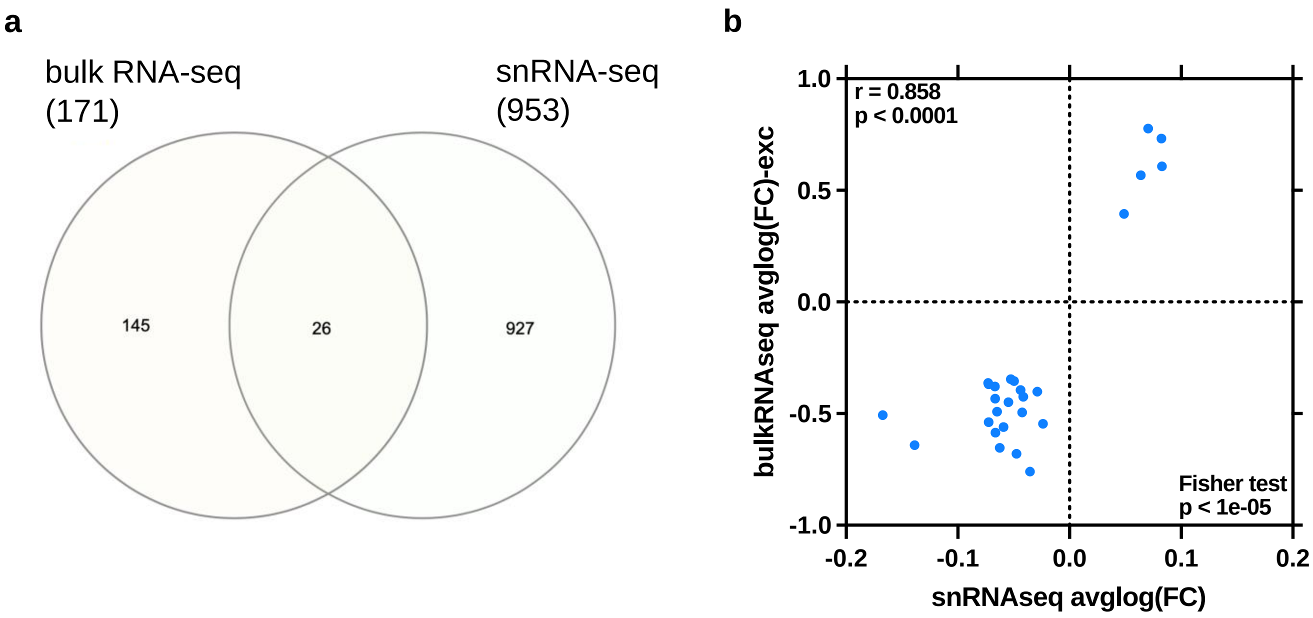

Figure S4. Comparison of the transcriptome changes revealed by bulk and snRNA-seq. a) Venn diagram showing the intersection between differentially expressed genes (adjusted p-value < 0.05) identified by bulk RNA-seq and detected in excitatory cells by snRNA-seq (Fisher test showing a significant overlap between the two groups, $p < 0.00001$). The numbers indicate the numerosity of the sets. **b)** Scatter plot correlating the expression changes in genes significantly regulated in bulk RNA-seq (logFC, y axis) and detected in excitatory cells by snRNA-seq (logFC, x axis) in adult mice (Spearman $r = 0.858$, $p < 0.0001$; Fisher test showing significant enrichment in the II and IV quadrants, $p < 1e-05$).

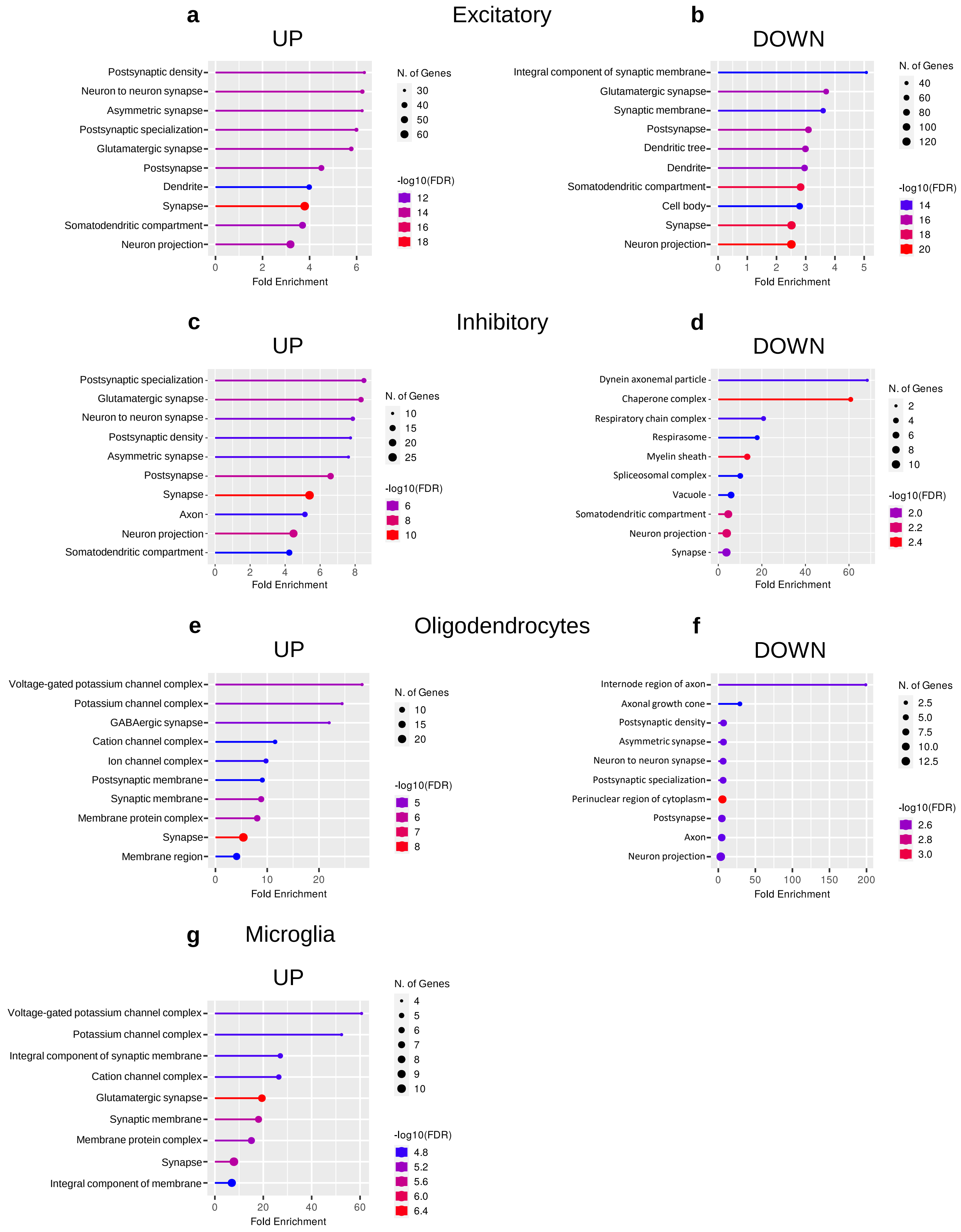

Excitatory
a
UP
Postsynaptic density
Neuron to neuron synapse
Asymmetric synapse
Postsynaptic specialization
Glutamatergic synapse
Postsynapse
Dendrite
Synapse
Somatodendritic compartment
Neuron projection
0 2 4 6
Fold Enrichment
N. of Genes
30
40
50
60
-log10(FDR)
12
14
16
18
b
DOWN
Integral component of synaptic membrane
Glutamatergic synapse
Synaptic membrane
Postsynapse
Dendritic tree
Dendrite
Somatodendritic compartment
Cell body
Synapse
Neuron projection
0 1 2 3 4 5
Fold Enrichment
N. of Genes
40
60
80
100
120
-log10(FDR)
14
16
18
20
Inhibitory
c
UP
Postsynaptic specialization
Glutamatergic synapse
Neuron to neuron synapse
Postsynaptic density
Asymmetric synapse
Postsynapse
Synapse
Axon
Neuron projection
Somatodendritic compartment
0 2 4 6 8
Fold Enrichment
N. of Genes
10
15
20
25
-log10(FDR)
6
8
10
d
DOWN
Dynein axonemal particle
Chaperone complex
Respiratory chain complex
Respirasome
Myelin sheath
Spliceosomal complex
Vacuole
Somatodendritic compartment
Neuron projection
Synapse
0 20 40 60
Fold Enrichment
N. of Genes
2
4
6
8
10
-log10(FDR)
2.0
2.2
2.4
Oligodendrocytes
e
UP
Voltage-gated potassium channel complex
Potassium channel complex
GABAergic synapse
Cation channel complex
Ion channel complex
Postsynaptic membrane
Synaptic membrane
Membrane protein complex
Synapse
Membrane region
0 10 20
Fold Enrichment
N. of Genes
10
15
20
-log10(FDR)
5
6
7
8
f
DOWN
Internode region of axon
Axonal growth cone
Postsynaptic density
Asymmetric synapse
Neuron to neuron synapse
Postsynaptic specialization
Perinuclear region of cytoplasm
Postsynapse
Axon
Neuron projection
0 50 100 150 200
Fold Enrichment
N. of Genes
2.5
5.0
7.5
10.0
12.5
-log10(FDR)
2.6
2.8
3.0
g
Microglia
UP
Voltage-gated potassium channel complex
Potassium channel complex
Integral component of synaptic membrane
Cation channel complex
Glutamatergic synapse
Synaptic membrane
Membrane protein complex
Synapse
Integral component of membrane
0 20 40 60
Fold Enrichment
N. of Genes
4
5
6
7
8
9
10
-log10(FDR)
4.8
5.2
5.6
6.0
6.4

Figure S5. Gene ontology (GO) analysis for cellular component from the snRNA-seq data set showing enrichment in synaptic components in neuronal and non-neuronal cell populations. Fold enrichment of top-level overrepresented GO terms in excitatory **(a,b)** and inhibitory **(c,d)** neurons, oligodendrocytes **(e,f)** and microglia **(g)**. Calculated by ShinyGO 0.76.3, FDR < 0.05.

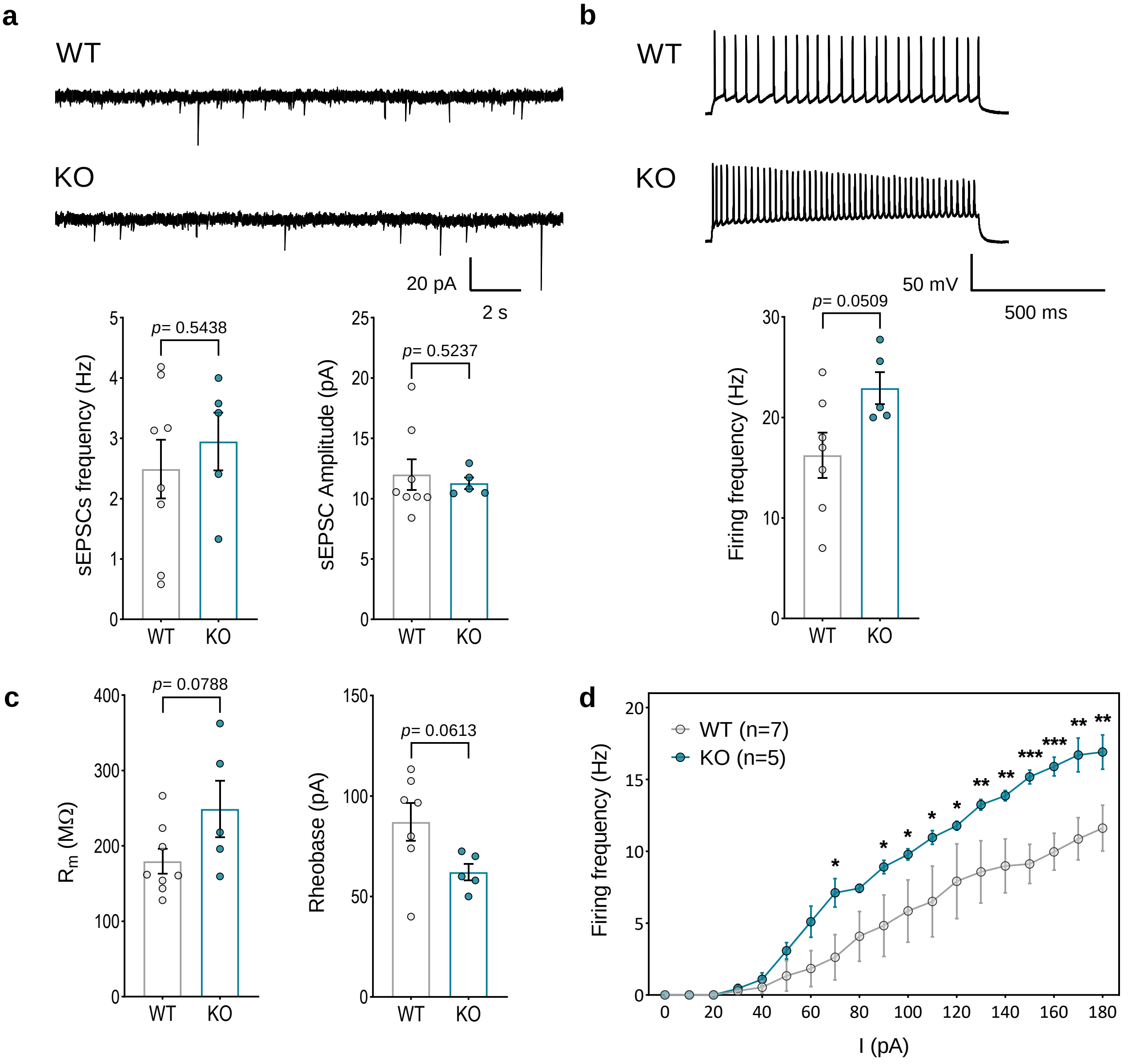

Figure S6. Electrophysiological characterization of pyramidal neurons in the PFC of $CrT^{+/y}$ (WT) and $CrT^{-/y}$ (KO) mice. Recordings were obtained from layer II/III pyramidal neurons from 8 $CrT^{+/y}$ and 5 $CrT^{-/y}$ animals at PND35-40, dots represent average values from each animal. Unless otherwise stated, measurements were performed on all subjects. a) Representative traces (top) and quantification (bottom) of spontaneous excitatory postsynaptic currents (sEPSCs) in pyramidal cells of $CrT^{+/y}$ and $CrT^{-/y}$ animals. No differences were found between the genotypes in frequency (t-test, $p= 0.5438$) and amplitude (Mann-Whitney test, $p= 0.5237$). b) Representative traces (top) and quantification (bottom) of firing frequency, showing a trend towards an increased firing in $CrT^{-/y}$ pyramidal neurons (t-test, $p= 0.0509$; n= 7 for $CrT^{+/y}$ and 5 for $CrT^{-/y}$). c) $CrT^{-/y}$ pyramidal neurons display a trend towards higher membrane resistance (left, t-test, $p= 0.0788$) and lower rheobase (right, t-test, $p= 0.0613$) compared to controls. d) Frequency vs. current plot showing persistently increased firing frequency in $CrT^{-/y}$ cells across a range of injected currents (Two-way RM ANOVA followed by Fisher's LSD test; n= 7 for $CrT^{+/y}$ and 5 for $CrT^{-/y}$). *$p< 0.05$, **$p< 0.01$, ***$p<0.001$. Data are expressed as mean ± SEM.

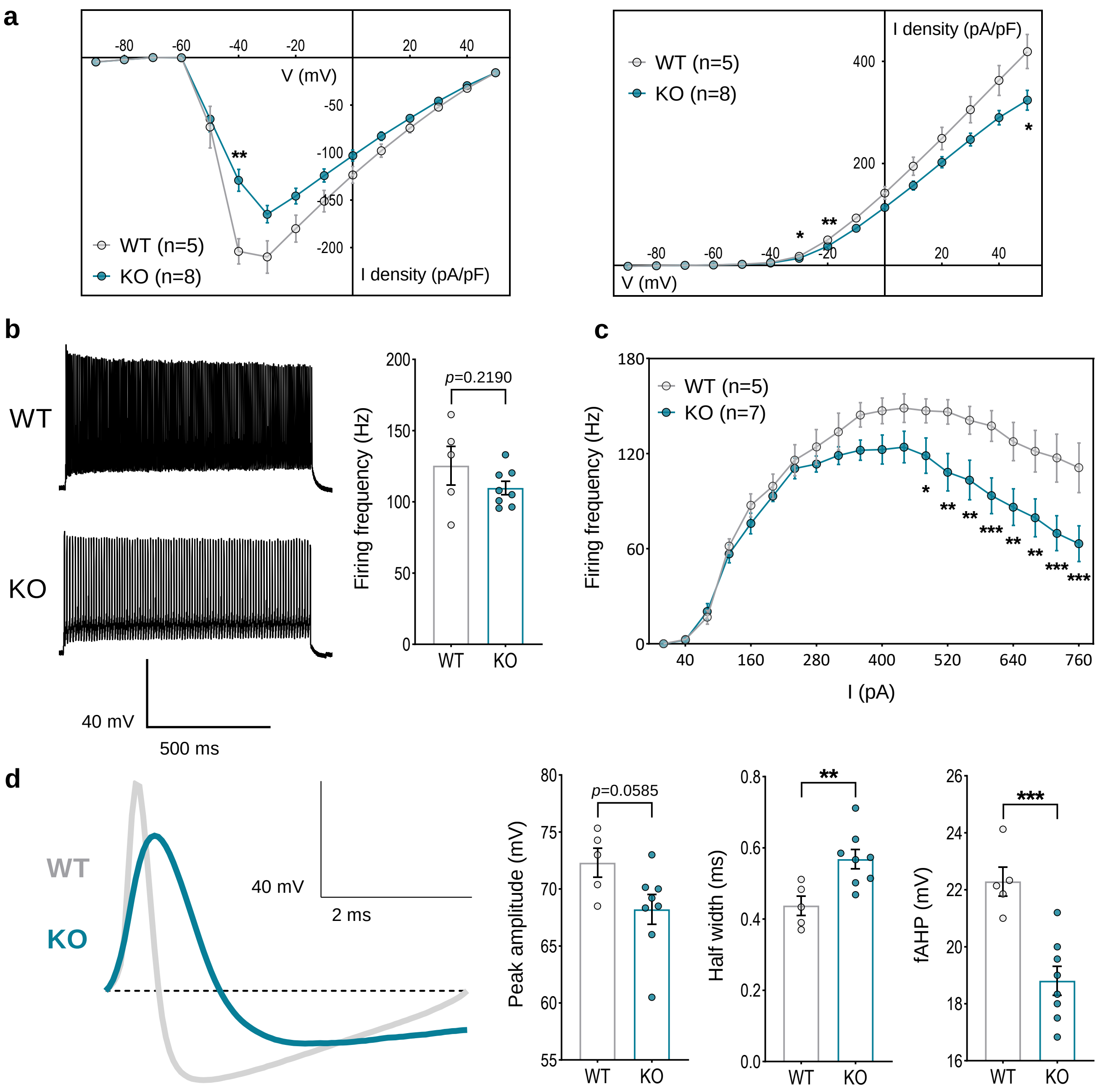
a
V (mV)
I density (pA/pF)
WT (n=5)
KO (n=8)
**
*
b
WT
KO
40 mV
500 ms
Firing frequency (Hz)
p=0.2190
c
I (pA)
WT (n=5)
KO (n=7)
d
WT
KO
40 mV
2 ms
Peak amplitude (mV)
p=0.0585
Half width (ms)
fAHP (mV)
***

Figure S7. Electrophysiological characterization of PV^+ interneurons in the PFC of $CrT^{+/y}$ (WT) and $CrT^{-/y}$ (KO) mice. Recordings were obtained from GFP^+ cells from 5 $CrT^{+/y}$ and 8 $CrT^{-/y}$ animals carrying the Cre-recombinase in PV^+ interneurons (PV::$CrT^{+/y}$ and PV::$CrT^{-/y}$), injected with the AAV9 pCAG-FLEX-EGFP-WPRE vector. Dots represent average values from each animal. Unless otherwise stated, measurements were performed on all subjects. a) Current-voltage relationships showing a decrease in peak Na^+ (left) and steady-state K^+ (right) current densities in PV::$CrT^{-/y}$ (KO) neurons with respect to controls (Two-way RM ANOVA followed by Fisher's LSD test). b) Representative traces (left) and quantification (right), showing a trend towards a decrease in firing frequency in PV::$CrT^{-/y}$ neurons (t-test, $p= 0.2190$). c) Frequency vs. current plot showing that PV::$CrT^{-/y}$ neurons persistently fire at lower frequency across a broad range of injected currents compared to controls (Two-way RM ANOVA followed by Fisher's LSD test; $n= 5$ for PV::$CrT^{+/y}$ and 7 for PV::$CrT^{-/y}$). d) Representative trace showing the typical profile of action potentials in the two groups (left) and quantification of action potential amplitude (left graph), half-width (central graph) and fAHP (right graph), indicating an alteration of all three parameters in $CrT^{-/y}$ neurons (t-test, $p= 0.0585$ for peak amplitude, $p <0.1$ for half-width, $p<0.001$ fAHP). *$p< 0.05$, **$p< 0.01$, ***$p<0.001$. Data are expressed as mean ± SEM.

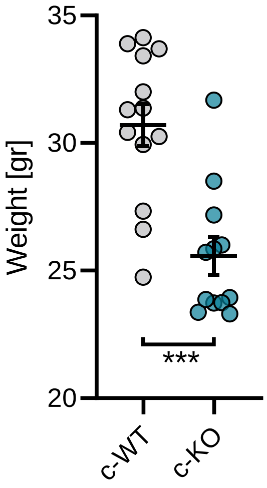

Figure S8. Weight difference between PV::CrTfl$^{+/y}$ (c-WT) and PV::CrTfl$^{-/y}$ (c-KO) animals at PND190. Lower weight of PV::CrTfl$^{-/y}$ (n= 12) compared to sex and age matched PV::CrTfl$^{+/y}$ controls (n= 13; t-test, $p < 0.001$). ***$p < 0.01$. Data are expressed as mean ± SEM. Circles represent individual data values.
